# Clonal evolution and clinical implications of genetic abnormalities in blastic transformation of chronic myeloid leukaemia

**DOI:** 10.1038/s41467-021-23097-w

**Published:** 2021-05-14

**Authors:** Yotaro Ochi, Kenichi Yoshida, Ying-Jung Huang, Ming-Chung Kuo, Yasuhito Nannya, Ko Sasaki, Kinuko Mitani, Noriko Hosoya, Nobuhiro Hiramoto, Takayuki Ishikawa, Susan Branford, Naranie Shanmuganathan, Kazuma Ohyashiki, Naoto Takahashi, Tomoiku Takaku, Shun Tsuchiya, Nobuhiro Kanemura, Nobuhiko Nakamura, Yasunori Ueda, Satoshi Yoshihara, Rabindranath Bera, Yusuke Shiozawa, Lanying Zhao, June Takeda, Yosaku Watatani, Rurika Okuda, Hideki Makishima, Yuichi Shiraishi, Kenichi Chiba, Hiroko Tanaka, Masashi Sanada, Akifumi Takaori-Kondo, Satoru Miyano, Seishi Ogawa, Lee-Yung Shih

**Affiliations:** 1 Department of Pathology and Tumor Biology, Graduate School of Medicine, Kyoto University, Kyoto, Japan; 2 Department of Hematology and Oncology, Graduate School of Medicine, Kyoto University, Kyoto, Japan; 3 Division of Hematology-Oncology, Department of Internal Medicine, Chang Gung Memorial Hospital-Linkou, Taoyuan, Taiwan; 4 College of Medicine, Chang Gung University, Taoyuan, Taiwan; 5 Department of Hematology and Oncology, Dokkyo Medical University, Tochigi, Japan; 6 Laboratory of Molecular Radiology, Center for Disease Biology and Integrative Medicine, Graduate School of Medicine, The University of Tokyo, Tokyo, Japan; 7 Department of Medical Genomics, Graduate School of Medicine, The University of Tokyo, Tokyo, Japan; 8 Department of Hematology, Kobe City Medical Center General Hospital, Kobe, Japan; 9 Department of Genetics and Molecular Pathology, Centre for Cancer Biology, SA Pathology, Adelaide, SA Australia; 10 Department of Haematology, Royal Adelaide Hospital and SA Pathology, Adelaide, SA Australia; 11 Department of Hematology, Tokyo Medical University, Tokyo, Japan; 12 Department of Hematology, Nephrology, and Rheumatology, Akita University Graduate School of Medicine, Akita, Japan; 13 Department of Hematology, Juntendo University School of Medicine, Tokyo, Japan; 14 Department of Hematology, Gifu University Hospital, Gifu, Japan; 15 Department of Hematology and Oncology, Kurashiki Central Hospital, Kurashiki, Japan; 16 Department of Hematology, Hyogo College of Medicine Hospital, Nishinomiya, Japan; 17 Institute for the Advanced Study of Human Biology (WPI-ASHBi), Kyoto University, Kyoto, Japan; 18 Laboratory of DNA Information Analysis, Human Genome Center, Institute of Medical Science, The University of Tokyo, Tokyo, Japan; 19 Department of Advanced Diagnosis, Clinical Research Center, National Hospital Organization Nagoya Medical Center, Nagoya, Japan; 20 Department of Medicine, Centre for Haematology and Regenerative Medicine, Karolinska Institute, Stockholm, Sweden

**Keywords:** Cancer genetics, Haematological cancer, Mutation

## Abstract

Blast crisis (BC) predicts dismal outcomes in patients with chronic myeloid leukaemia (CML). Although additional genetic alterations play a central role in BC, the landscape and prognostic impact of these alterations remain elusive. Here, we comprehensively investigate genetic abnormalities in 136 BC and 148 chronic phase (CP) samples obtained from 216 CML patients using exome and targeted sequencing. One or more genetic abnormalities are found in 126 (92.6%) out of the 136 BC patients, including the *RUNX1*-*ETS2* fusion and *NBEAL2* mutations. The number of genetic alterations increase during the transition from CP to BC, which is markedly suppressed by tyrosine kinase inhibitors (TKIs). The lineage of the BC and prior use of TKIs correlate with distinct molecular profiles. Notably, genetic alterations, rather than clinical variables, contribute to a better prediction of BC prognosis. In conclusion, genetic abnormalities can help predict clinical outcomes and can guide clinical decisions in CML.

## Abstract (web summary)

In chronic myeloid leukaemia (CML), the drivers of blast crisis and resistance to tyrosine kinase inhibitors are not fully characterised. Here, the authors analyse a cohort of CML samples with genomic technologies and find that at least one driver alteration is associated with progression and worse prognosis.

## Introduction

Chronic myeloid leukaemia (CML) is a myeloproliferative disorder caused by the *BCR*-*ABL1* gene fusion generated in the Philadelphia (Ph) chromosome, der(22)t(9;22)(q34;q11.2). Recently, the prognosis of CML has been dramatically improved by the development of tyrosine kinase inhibitors (TKIs) targeting the BCR-ABL1 fusion protein. However, a minority of patients in the chronic phase (CP) fail to respond to TKI therapy, progress to blast crisis (BC), and show dismal clinical outcomes^1^. While a mutation in the BCR-ABL1 kinase domain is known to be one of the major determinants of TKI resistance and a risk for blastic transformation^2^, additional genetic alterations have been hypothesised to be necessary for the progression to BC. In fact, recent studies have demonstrated several driver mutations acquired during blastic transformation^3–17^. However, the current understanding of the genetic basis of TKI resistance, and progression of CML-CP to BC remains limited by the small number of patients and/or genes analysed in each study, as well as the paucity of matched CP and BC samples.

Another point of interest is the improved 5-year overall survival (OS) of BC patients from 16% during 2000–2004 to 33% during 2010–2016, which may be attributed to increased use of TKIs^18^. TKIs may be effective for certain patients with BC and contribute to improved survival; however, the majority of BC patients no longer show response to TKIs. If this is indeed the case, it is of considerable clinical importance to predict which BC patients can respond to TKI, for better management of CML. Unfortunately, only a few clinical factors or biomarkers are currently known to be correlated with clinical outcomes of BC patients treated with TKI-based regimens.

In this study, we investigated a large cohort of CML patients to reveal the landscape of genetic lesions in CML during both CP and BC. We aimed to identify genetic alterations that correlated with TKI-resistance and blastic transformation, as well as those that predicted clinical outcomes. For this, genetic alterations in both CP and BC samples, including paired CP and BC samples, were analysed using unbiased sequencing.

## Results

### Clonal evolution of CML

**Fig. 1:**
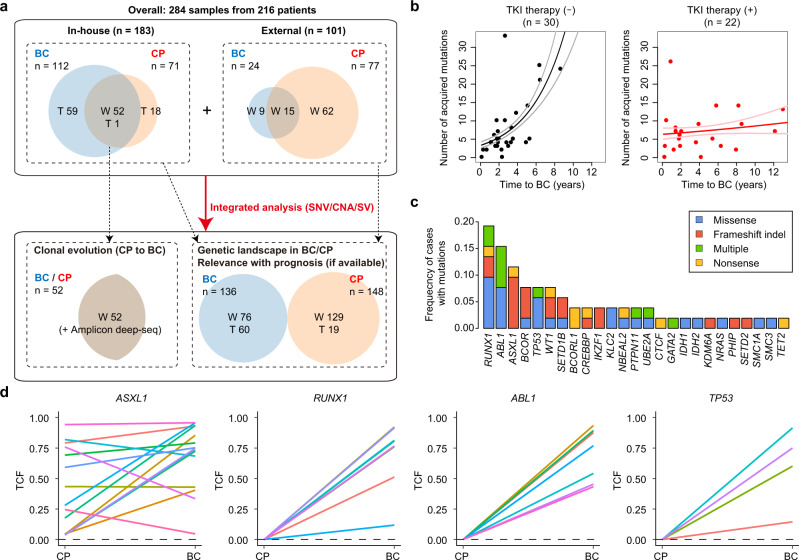
Somatic mutations acquired during clonal evolution in CML. **a** Scheme demonstrating the study cohort. Numbers indicate analysed samples. Numbers in overlapping regions of CP and BC circles indicate the number of cases analysed for both CP and BC. W whole-exome sequencing, T targeted capture sequencing. **b** Scatter plots for time to progression (horizontal axis) and number of acquired SNVs (vertical axis) during progression from CP to BC in 52 cases for whom WES was performed, using paired CP and BC samples. Regression lines with 95% confidence intervals were also plotted. Cases with or without TKI therapy after CP diagnosis are indicated separately. **c** Frequencies of mutations acquired during evolution from CP to BC in 52 cases. Recurrently acquired or known driver genes are described. Categories of mutations are depicted in different colours, and “multiple” indicates ≥2 distinct mutations found in the same gene in the same patient. **d** TCFs of the indicated mutations in the corresponding CP and BC samples determined by conducting deep amplicon sequencing. Black dashed lines indicate a TCF of 0%. Colours represent individual cases.

First, we performed whole-exome sequencing (WES) of paired CP and BC samples obtained from 52 patients with CML, to identify genetic alterations that were relevant to the clonal evolution to CML-BC, at a mean depth of 157 (Fig. 1a, Supplementary Fig. 1a, and Supplementary Table 1). On average, 5.3 (0–21) nonsynonymous single-nucleotide variants (SNVs; or 0.088/Mb) were acquired during disease progression from CP to BC, with a median time of 26.7 months (0.7–155.1; Fig. 1b and Supplementary Fig. 1b). Notably, a Poisson regression model revealed that the number of mutations acquired during progression from CP to BC was independently and positively correlated with the interval between the progression (*P* = 9.4 × 10^−12^), and negatively correlated with TKI therapy after CP diagnosis (*P* = 9.3 × 10^−3^; Fig. 1b). The correlation with the number of recurrent mutations in CML-BC was not clear (Supplementary Fig. 1c). A similar trend was observed in a previous cohort of paired samples^13^, even though our findings did not show statistical significance owing to the small number of evaluable samples after quality control (*n* = 13; Supplementary Fig. 1d). These results suggest that transformation from CP to BC is associated with accumulation of somatic mutations with time in the absence of effective therapy, and this accumulation is noticeably suppressed by TKI therapy, which may prevent the transformation from CP to BC.

In the CML-BC samples, mutations were frequently found in the driver genes implicated in myeloid malignancies, including *RUNX1*, *ABL1*, *ASXL1*, *BCOR*/*BCORL1*, *TP53*, and *WT1* (Fig. 1c and Supplementary Fig. 1e). We also identified recurrent mutations in recently reported genes in BC, such as *UBE2A*^13,14^ and *SETD1B*^13^, as well as in previously unreported genes, such as *KLC2* and *NBEAL2*. Deep amplicon sequencing of these mutations at a mean depth of ×2589 in paired CP and BC samples revealed that *ASXL1* mutations were already present in the CP samples, whereas other major drivers, including *RUNX1*, *ABL1*, and *TP53* mutations were initially absent in CP and emerged during progression to BC (Fig. 1d). In a few patients, mutations in other genes, such as *WT1* and *IDH2*, were also found in the corresponding CP samples with lower tumour cell fractions (TCFs) calculated by variant allele frequencies (VAFs) than those found in the BC samples (Supplementary Fig. 1f). These results suggest distinct roles for the different mutations in the progression of CML. TCFs of *ASXL1* mutations were increased in nine, decreased in three, and almost stable (<10% difference) in three patients during disease progression from CP to BC. Almost all patients with *ASXL1* mutations showed acquisition of other additional genetic abnormalities during progression to BC (93.3%, 14 out of 15 cases), including mutations (12/15) in *RUNX1* (4/15), *TP53* (3/15), *BCOR* (2/15), and *SETD1B* (2/15) genes. Typically, at least one accompanying mutation had TCFs comparable to *ASXL1* mutations and was probably present in the major clones in the BC samples (Supplementary Fig. 2). Therefore, *ASXL1*-mutated CP clones may be preferentially selected and may evolve by acquiring other drivers during the clonal development to BC.

**Fig. 2:**
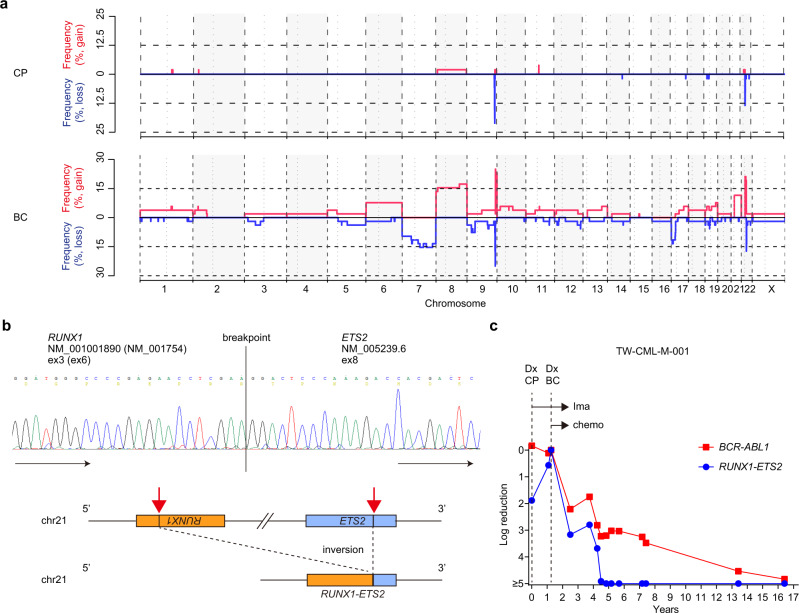
CNAs and *RUNX1*-*ETS2* fusion in CML-CP and BC. **a** Sequencing-based copy-number profiling in CML-CP and BC samples from 52 cases. Frequencies of copy-number gains or losses are depicted along the chromosomal regions. **b** *RUNX1*-*ETS2* fusion detected in a patient (TW-CML-M-001) by conducting WES analysis. Sanger sequencing of cDNA obtained from the BC samples was used to validate the fusion breakpoint (top). Simplified scheme of *RUNX1*-*ETS2* fusion (bottom). Inversion 21 involves the *RUNX1* and *ETS2* genes for generation of the fusion. The karyotyping result of this sample at CP diagnosis was as follows (results at BC diagnosis were not obtained): 47,XY,+8,inv(9)(p11q13),t(9;22)(q34;q11). **c** Time chart showing dynamic changes in *RUNX1-ETS2* and *BCR-ABL1* burdens assessed by RT-qPCR at several time points in a patient (TW-CML-M-001). The horizontal and vertical axes represent the time from CP diagnosis (Dx) and levels of the indicated transcripts, respectively. The patient received imatinib (Ima) treatment after CP diagnosis and achieved CHR, while the *BCR*-*ABL1* burden was not reduced significantly. Approximately 15 months after CP diagnosis, there was an abrupt development of BC, which was successfully treated with cytarabine and hydroxyurea in addition to imatinib. Thereafter, *BCR-ABL1* transcript levels showed continuous reductions, and were below the threshold of CMR at 15 years after BC diagnosis. The *RUNX1*-*ETS2* transcript was detected at the time of CP diagnosis and increased markedly during BC development, declined following chemotherapy with imatinib, and was undetectable 4 years after BC transformation.

We also performed sequencing-based copy-number analysis^19^. Copy-number alterations (CNAs) were frequently found in BC, but were rarely present in CP (Fig. 2a), suggesting that CNAs were also acquired during progression from CP to BC. Frequently identified CNAs in BC included −7/del(7p), +8, del(17p), amp(17q), +21, and an extra Ph chromosome (+Ph). We also analysed structural variations (SVs) close to the gene bait regions based on an algorithm, utilising both breakpoint-containing junction read pairs and improperly aligned read pairs^20^. Although the ability to detect SVs in our pipeline depends on the location of breakpoints and gene baits (see “Methods”), this approach led to the identification of an inversion event, resulting in a *RUNX1*-*ETS2* fusion in a patient with myeloid BC, which was confirmed by performing reverse transcription PCR (RT-PCR) and Sanger sequencing (Fig. 2b and Supplementary Fig. 3). Quantitative RT-PCR (RT-qPCR) at several time points demonstrated that this fusion was already present at the time of CP diagnosis, and the burden of this fusion, which was reduced after successful chemotherapy for CML-BC, was correlated with that of *BCR*-*ABL1* (Fig. 2c). Thus, *RUNX1*-*ETS2* may play a role in the rapid progression of CML-CP to BC.

### Genetic landscape of CML-BC

**Fig. 3:**
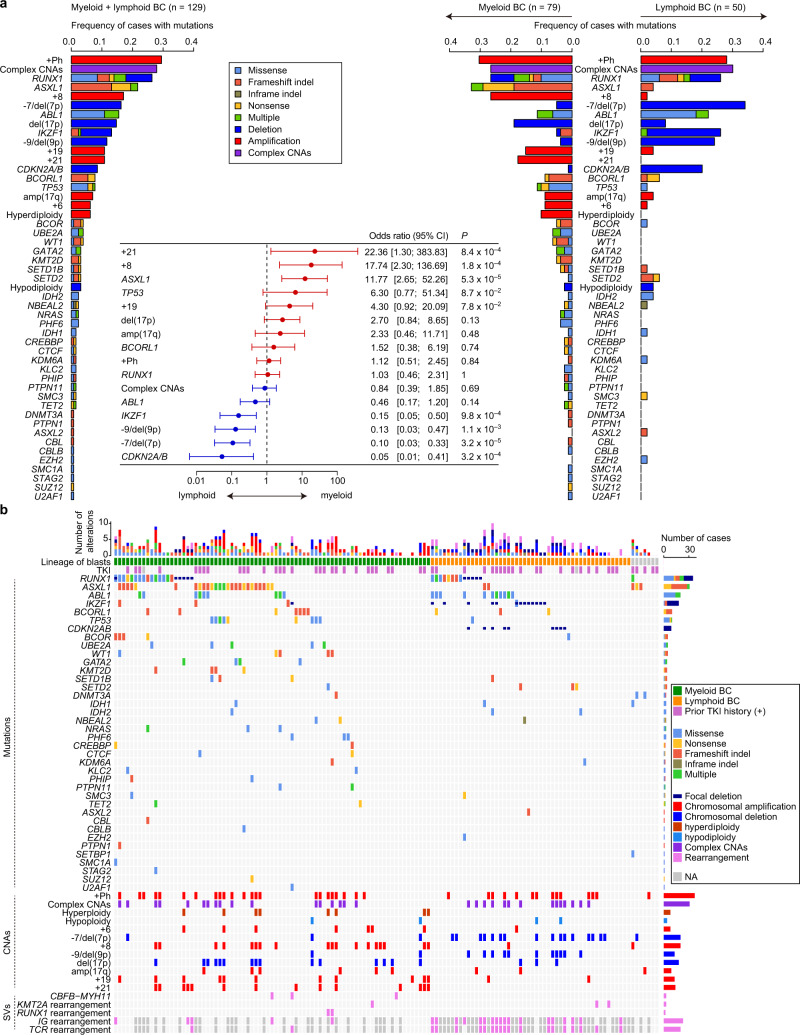
Genetic landscape of myeloid and lymphoid BC in CML. **a** Frequencies of mutations in 129 BC patients evaluable for blast lineage (left panel), and myeloid (*n* = 79) and lymphoid BC (*n* = 50) (right panel). Categories of mutations are depicted in different colours, and “multiple” indicates ≥2 distinct alterations found in the same gene in the same patient. The forest plot shows odds ratios with 95% confidence intervals (CI) for enrichment of each genetic lesion in myeloid BC. The dashed line represents an odds ratio of 1. Positive and negative odds ratios are indicated by red and blue colours, respectively. Genetic lesions found in >10 cases were included. *P* values were calculated using the Fisher’s exact test. **b** Summary of genetic lesions in all 136 BC patients. Each column indicates one patient. Lineage of blasts and prior history of TKI therapy before BC diagnosis are also shown. Categories of alterations are depicted in different colours, and “multiple” indicates ≥2 distinct mutations found in the same gene in the same patient. The rearrangement of immunoglobulin (*IG*) and T-cell receptor (*TCR*) genes is shown in cases analysed by WES. NA not available.

Next, we performed targeted capture sequencing that covered 104 myeloid tumour-associated genes, including candidates of drivers found by WES in an additional 60 BC and 19 CP samples at a mean depth of ×585 (Supplementary Table 2). Combined with the WES data from the 52 paired BC and CP samples, as well as external WES data for 24 BC^12,13^ and 77 CP^13,21,22^ patients, we comprehensively analysed a total of 136 BC and 148 CP cases for SNVs, CNAs, and SVs (Fig. 1a and Supplementary Table 1). Clinical information of the external data of patients was typically limited to a few factors, including age, TKI therapy and response, and lineage of BC blasts (Table 1 and Supplementary Table 3). Myeloid and lymphoid crises accounted for 62.8% and 37.2% of the total cohort, respectively. In addition, 42.5% of the cohort presented with a prior history of TKI therapy before BC diagnosis, where in most cases, the initial TKI used was imatinib (94.3%). In total, 126 out of 136 patients with CML-BC harboured at least one mutation or CNA (Fig. 3, Supplementary Figs. 4 and 5, Supplementary Table 4, and Supplementary Data 1). Most mutations (89.7%, 192/214) involved genes implicated in epigenetic regulation and signalling, such as chromatin modification, DNA methylation, transcription factors, the cohesin complex, and signalling pathways. In addition to *KLC2* and *NBEAL2*, we found another recurrent mutational target, *PHIP*, which was mutated in two patients. Sequencing-based copy-number analysis disclosed the presence of complex CNAs (defined as ≥3 abnormal CNAs) in 26.5% of the BC patients, although complex karyotypes in CML-BC have previously been reported at a lower rate (10–12%) based on conventional karyotyping^23^, whose resolution is relatively limited.

**Table 1:** Characteristics of patients with CML-BC.

Number of cases	136
Age at BC diagnosis (y), median (range) (*n* = 126)	50 (16–86)
Sex, *n* (%) (*n* = 132)	
Male	79 (59.8)
Female	53 (40.2)
Lineage of blasts, *n* (%) (*n* = 129)	
Myeloid	81 (62.8)
Lymphoid	48 (37.2)
WBC (×10^3^/uL), median (range) (*n* = 123)	40,600 (1700–580,000)
Hb (g/dL), median (range) (*n* = 123)	9.6 (5.0–15.8)
PLT (×10^3^/uL), median (range) (*n* = 123)	105,000 (3000–2,740,000)
LDH (U/L), median (range) (*n* = 79)	696 (75–6332)
Blasts in BM (%), median (range) (*n* = 126)	59.5 (1.0–98.4)
Prior history of CP diagnosis, *n* (%) (*n* = 135)	
Yes	103 (76.3)
No	32 (23.7)
Time from CP diagnosis (m), median (range) (*n* = 93)	34.3 (0.27–363)
Age at CP diagnosis (y), median (range) (*n* = 92)	45 (14–85)
Prior TKI before BC, *n* (%) (*n* = 134)	
Yes	57 (42.5)
No	77 (57.5)
TKIs used for CP, *n* (%) (*n* = 53)	
Imatinib	50 (94.3)
Dasatinib	2 (3.8)
Nilotinib	1 (1.9)
TKI-based therapy for BC, *n* (%) (*n* = 119)	
Yes	72 (60.5)
No	47 (39.5)
TKIs used for BC, *n* (%) (*n* = 72)	
Imatinib	36 (50.0)
Dasatinib	32 (44.4)
Nilotinib	2 (2.8)
Ponatinib	2 (2.8)
Final status, *n* (%) (*n* = 123)	
Alive	26 (21.1)
Dead	97 (78.9)
Method, *n* (%) (*n* = 136)	
Whole-exome sequencing	76 (55.9)
Targeted capture sequencing	60 (44.1)
Median follow-up time (m), median (range) (*n* = 123)	38.2 (0.13–365)

We further explored the relationship between the genetic abnormalities and the lineage of BC (Fig. 3). Certain lesions, such as those attributed to +21, +8, +19, and *ASXL1* and *TP53* mutations, were enriched in myeloid crisis compared to those in lymphoid crisis, while others were enriched in lymphoid BC, which included *CDKN2A/B* and *IKZF1* deletions, −7/del(7p), and −9/del(9p). In contrast, abnormalities such as *RUNX1* mutations and +Ph were almost equally observed in both crises. The rearrangement of immunoglobulin and/or T-cell receptor genes, which was assessed by WES, was confirmed in most evaluable lymphoid samples and in a few samples of myeloid crisis. Analysis of pairwise correlations between the genetic lesions showed the existence of co-occurring patterns depending on the combination of lesions (Supplementary Fig. 6). Among these, the most conspicuous correlations were those observed between +6, +8, +19, and +21, which were highly specific to myeloid crisis. Another visible correlation was between −7/del(7p), −9/del(9p), and *CDKN2A/B* deletions, which were highly specific to lymphoid crisis. We also found that del(17p) co-occurred with *TP53* mutations, +Ph, +8, and amp(17q). Taken together, although substantial overlaps in genetic lesions were observed, myeloid and lymphoid BC cases were distinct in terms of lineage commitment, as well as their molecular profiles.

We next investigated whether a prior history of TKI therapy influenced the genetic profile in BC, whereby we compared genetic alterations in BC patients with and without a prior history of TKI therapy. As expected, *ABL1* mutations were almost exclusively found in patients who had received TKIs, representing the most frequent mutation (Supplementary Fig. 7). All *ABL1* mutations were accompanied by other genetic alterations, such as mutations in *RUNX1* (7/20), *ASXL1* (5/20), and *BCORL1* (5/20), focal deletions in *CDKN2A/B* (6/20), and *IKZF1* (4/20), and cytogenetic abnormalities, such as −7/del(7p) (6/20), −9/del(9p) (6/20), and +8 (4/20), which was consistent with previous reports^24^. Moreover, BC patients with a history of TKI treatment were more likely to harbour −7/del(7p), −9/del(9p), and complex CNAs than those without TKI treatment, while *BCOR*, *TP53*, and *RUNX1* mutations were less frequent in TKI-treated patients. This suggests that genetic profiles in CML-BC differ between TKI-treated and untreated patients, although it remains unclear whether non-*ABL1* mutations can affect TKI response/resistance.

### Prognostic relevance of genetic abnormalities in CML-BC

**Fig. 4:**
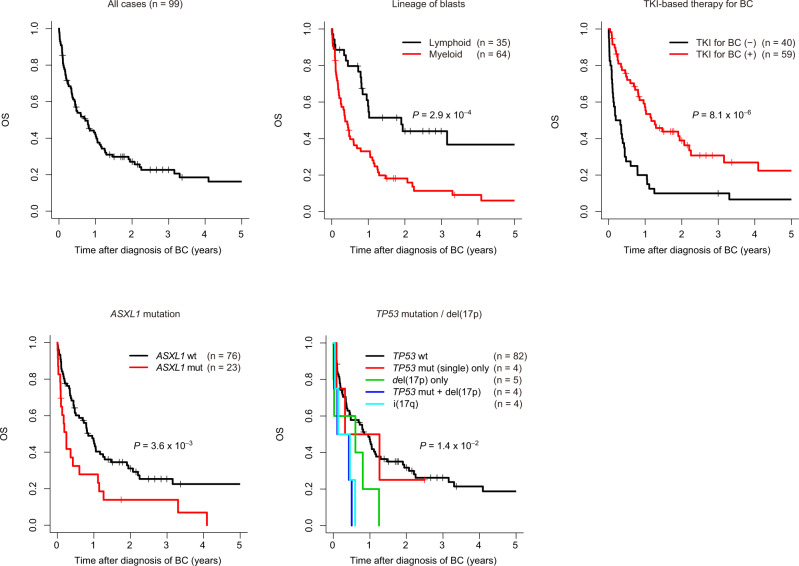
Prognostic relevance of genetic abnormalities in CML-BC. Kaplan–Meier survival curves for OS in 99 patients with CML-BC according to the indicated clinical or genetic factors. The prognostic impact of each factor on OS was calculated using the log-rank test. For *TP53* mutations and/or del(17p), we classified patients according to the presence or absence of *TP53* mutations, del(17p), and i(17q), based on which patients were divided into the following five groups: patients (1) without any *TP53* mutations/del(17p)/i(17q), (2) with *TP53* mutations alone, (3) with del(17p) alone, (4) with concurrent *TP53* mutations and del(17p), and (5) with i(17q). One patient harboured two distinct *TP53* mutations, as well as del(17p), and was classified into group 4. No patients with i(17q) harboured *TP53* mutations.

**Fig. 5:**
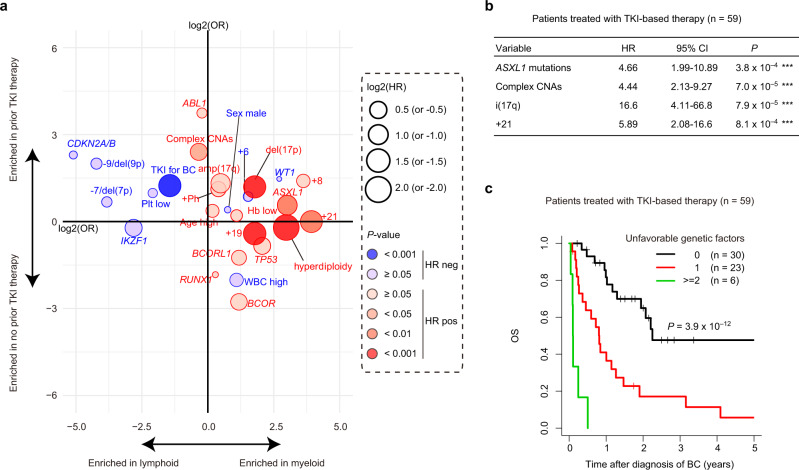
Prognostic impact of each clinical or genetic factor in CML-BC. **a** Summary of the relationship between individual clinical or genetic factors and lineage of BC, prior TKI history, and OS in 99 BC patients. Odds ratios for the enrichment of individual factors in myeloid lineage of BC and in the history of prior TKI therapy are represented in the horizontal and vertical axes, respectively. The size of the circles corresponds to the hazard ratio (HR) of the effect of each factor on the OS in the univariate analysis. Positive and negative HR values are shown as red and blue circles, respectively. The colour gradient indicates *P* values calculated by the Cox proportional hazards regression model in the univariate analysis. **b** Cox proportional hazards regression model with a stepwise variable selection identifying the independent risk factors, predicting OS in 59 patients treated with TKI-based therapy for BC. ***P < 0.001. **c** Kaplan–Meier survival curves for OS of 59 patients treated with TKI-based therapy for BC according to the number of unfavourable genetic factors listed in **b**. *P* values were calculated using the log-rank test.

We next analysed the prognostic relevance of the genetic abnormalities, as well as the clinical features in 99 CML-BC patients for whom survival information was available. With a median follow-up of 3.2 years (0.48–30.4), the estimated 2-year OS rate for all patients was 27.1% (95% confidence interval, 19.3–38.0; Fig. 4 and Supplementary Table 5). In the univariate analysis of clinical features for OS, TKI therapy for BC and lineage of blasts were significantly associated with OS, while other parameters, including age, sex, blood counts, and TKI history before BC, did not exhibit significant associations (Figs. 4 and 5a, and Table 2). Univariate analysis of genetic lesions observed in >5% of the patients revealed a negative prognostic impact of *ASXL1* mutations, del(17p), i(17q) (isochromosome 17q, resulting in one copy of 17p and three copies of 17q), +19, +21, hyperdiploidy (as defined by presence of ≥48 chromosomes assessed by sequencing-based copy-number profiling), and complex CNAs (Table 2). Conspicuously, patients with concurrent *TP53* mutations and del(17p), which were predictive of biallelic targeting of *TP53*, and i(17q), showed an especially grim outcome (Fig. 4). Consistent with a previous report demonstrating the association of multiple-hit *TP53* mutations with complex karyotypes and poor outcomes in myelodysplastic syndromes^25^, three out of four cases with biallelic *TP53* mutations in CML-BC patients revealed the existence of a complex karyotype. After performing adjustment for blast lineage and TKI-based therapy to correct for the possible effects of an association between the clinical and genetic factors, complex CNAs, −7/del(7p), and amp(17q) showed significant association with OS (Table 2 and Supplementary Fig. 8). As shown in Fig. 5a, several genetic lesions were considerably and strongly associated with the lineage of BC, prior TKI-based treatment history, and/or prognosis in CML-BC, compared with clinical features, such as age, sex, white blood cell (WBC) count, haemoglobin levels, and platelet count. Therefore, genetic abnormalities may be good biomarkers for predicting clinical outcomes in BC patients.

**Table 2:** Univariate analysis for OS of CML-BC.

Variable	All, *n* = 99							TKI treated, *n* = 59						
	Univariate				Adjusted			Univariate				Adjusted		
	*n*	HR	95% CI	*P*	HR	95% CI	*P*	*n*	HR	95% CI	*P*	HR	95% CI	*P*
Clinical factors														
Age at BC (>60 y)	27	1.26	0.76–2.10	0.36	1.89	1.08–3.31	2.5 × 10^−2^*	19	1.34	0.68–2.63	0.40	1.35	0.68–2.67	0.39
Sex (male)	59	0.98	0.62–1.55	0.92	1.01	0.63–1.62	0.97	37	0.85	0.44–1.63	0.62	0.87	0.45–1.68	0.68
WBC (>50,000 × 10^3^/uL)	50	0.77	0.49–1.23	0.27	0.73	0.46–1.17	0.19	27	0.50	0.26–0.96	3.6 × 10^−2^*	—	—	—
Hb (<9.3 g/dL)	49	1.19	0.75–1.87	0.46	0.79	0.49–1.27	0.32	25	1.20	0.63–2.27	0.58	1.07	0.55–2.08	0.85
Plt (<96,000 × 10^3^/uL)	49	0.92	0.58–1.44	0.70	1.38	0.83–2.27	0.21	30	1.16	0.61–2.22	0.64	1.20	0.60–2.41	0.61
Lineage of blasts (myeloid)	64	2.59	1.53–4.39	3.8 × 10^−4^***	—	—	—	33	2.08	1.06–4.07	3.3 × 10^−2^*	—	—	—
Prior history of TKI before BC	36	0.93	0.58–1.51	0.78	1.05	0.64–1.73	0.84	26	1.41	0.74–2.70	0.29	0.70	0.32–1.56	0.39
TKI-based therapy for BC	59	0.36	0.23–0.58	2.0 × 10^−5^***	—	—	—	59	—	—	—	—	—	—
Mutations														
* ABL1*	13	1.11	0.58–2.11	0.75	1.17	0.62–2.23	0.63	8	1.55	0.67–3.55	0.30	1.09	0.46–2.59	0.85
* ASXL1*	23	2.09	1.26–3.47	4.4 × 10^−3^**	1.30	0.76–2.25	0.34	11	2.31	1.08–4.91	3.0 × 10^−2^*	2.61	1.07–6.37	3.4 × 10^−2^*
* BCOR*	5	1.58	0.63–3.96	0.33	1.75	0.69–4.42	0.24	4	2.99	1.03–8.70	4.4 × 10^−2^*	2.57	0.84–7.85	9.7 × 10^−2^
* BCORL1*	5	1.41	0.57–3.49	0.46	1.34	0.53–3.41	0.54	3	1.44	0.44–4.72	0.55	0.90	0.27–3.07	0.87
* CDKN2A/B*	7	0.95	0.41–2.19	0.91	1.84	0.70–4.87	0.22	5	1.97	0.75–5.17	0.17	2.82	0.87–9.13	8.4 × 10^−2^
* IKZF1*	12	0.61	0.27–1.41	0.25	1.03	0.42–2.53	0.95	10	1.09	0.42–2.81	0.86	1.87	0.65–5.35	0.24
* RUNX1*	28	1.02	0.63–1.67	0.93	1.06	0.64–1.76	0.81	21	1.45	0.76–2.77	0.26	1.68	0.86–3.27	0.13
* TP53*	8	1.62	0.74–3.53	0.23	1.46	0.66–3.22	0.35	3	0.90	0.22–3.75	0.88	0.80	0.19–3.36	0.76
CNAs														
+Ph	26	1.44	0.87–2.36	0.16	1.28	0.78–2.12	0.33	14	1.71	0.84–3.46	0.14	1.46	0.72–2.97	0.29
Complex CNAs	28	1.65	1.02–2.67	4.3 × 10^−2^*	1.66	1.00–2.73	4.8 × 10^−2^*	16	2.99	1.55–5.78	1.1 × 10^−3^**	3.37	1.69–6.69	5.3 × 10^−4^***
Hyperdiploidy	6	4.38	1.84–10.5	8.7 × 10^−4^***	2.28	0.93–5.57	7.0 × 10^−2^	1	10.99	1.28–94.1	2.9 × 10^−2^*	5.90	0.67–51.7	0.11
+6	6	0.91	0.33–2.52	0.86	0.75	0.27–2.08	0.58	2	0.63	0.08–4.65	0.65	0.21	0.03–1.68	0.14
−7/del(7p)	13	0.89	0.46–1.74	0.74	2.41	1.06–5.45	3.5 × 10^−2^*	9	0.84	0.35–2.01	0.69	1.63	0.56–4.72	0.37
+8	18	1.29	0.72–2.32	0.39	0.91	0.50–1.65	0.76	8	1.08	0.42–2.80	0.88	0.46	0.16–1.32	0.15
−9/del(9p)	9	0.87	0.40–1.89	0.72	1.30	0.52–3.25	0.57	5	1.97	0.75–5.17	0.17	2.82	0.87–9.13	8.4 × 10^−2^
del(17p)	13	2.78	1.50–5.18	1.2 × 10^−3^**	1.78	0.94–3.38	7.6 × 10^−2^	5	4.54	1.63–12.6	3.8 × 10^−3^**	2.86	0.97–8.44	5.6 × 10^−2^
amp(17q)	7	1.98	0.90–4.34	8.8 × 10^−2^	2.44	1.10–5.45	2.9 × 10^−2^*	5	2.59	1.00–6.73	5.0 × 10^−2^	2.62	0.98–6.99	5.5 × 10^−2^
i(17q)	4	2.81	1.00–7.84	4.9 × 10^−2^*	2.79	0.98–7.91	5.4 × 10^−2^	3	5.50	1.58–19.2	7.5 × 10^−3^**	2.89	0.78–10.7	0.11
+19	13	2.85	1.54–5.27	8.4 × 10^−4^***	1.66	0.87–3.18	0.12	4	4.48	1.49–13.5	7.5 × 10^−3^**	3.09	0.98–9.71	5.3 × 10^−2^
+21	11	2.63	1.37–5.05	3.6 × 10^−3^**	1.69	0.87–3.31	0.12	5	3.93	1.49–10.4	5.7 × 10^−3^**	3.23	1.16–8.97	2.4 × 10^−2^*

Univariate analysis for OS by Cox proportional hazard regression model. For all cases (*n* = 99), values adjusted for lineage of blasts and TKI-containing therapy for BC are also shown. For TKI treated cases (*n* = 59), values adjusted for WBC and lineage of blasts are also shown. Information for prior history of TKI is missing in two patients treated by TKI for BC.

*95% CI* 95% confidence interval, HR hazard ratio.

*P < 0.05; **P < 0.01; ***P < 0.001.

We next analysed survival, focusing on 59 TKI-treated patients, because TKI-based therapy has been shown to significantly improve OS and thus, is the current therapy of choice for patients with CML-BC^1,18,26^. In the univariate analysis, WBC count, lineage of blasts, *ASXL1* and *BCOR* mutations, complex CNAs, del(17p), i(17q), +19, and +21 were significantly associated with OS (Supplementary Fig. 9a). We also evaluated the relative effects of genetic alterations using Cox proportional hazard regression modelling with a standard backward selection of clinical and genetic variables, and identified *ASXL1* mutations, complex CNAs, i(17q), and +21 as independent predictors of worse prognosis (Fig. 5b). To internally validate this finding, modelling was performed 100 times by conducting the bootstrap, in which all four variables of the final model were selected at a frequency of >70%, with a mean concordance statistic of 0.74 (Supplementary Fig. 9b). Based on the number of these unfavourable factors, TKI-treated BC patients were classified into three subgroups showing distinct prognosis, where the 2-year OS rate was 65.0%, 17.1%, and 0% for patients with 0, 1, and ≥2 unfavourable genetic risk factors, respectively (*P* = 3.9 × 10^−12^; Fig. 5c). We also analysed an independent external cohort reported in a recent publication^13^, in which 17 CML-BC patients were evaluable for survival with 12 receiving TKI-based therapy. Although the number of cases was limited, several similar associations were observed in the external cohort. TKI-based therapy was associated with better OS, while *ASXL1* mutations and i(17q) predicted poor prognosis (Supplementary Fig. 10a). Even though the difference was not statistically significant, owing to the small number of samples analysed, patients with genetic risk factors tended to show a poor prognosis (Supplementary Fig. 10b). Therefore, our results suggest that genetic risk factors may help identify a subset of patients, who may be refractory to TKI therapy.

### Genetic landscape and clinical outcome of CML-CP

**Fig. 6:**
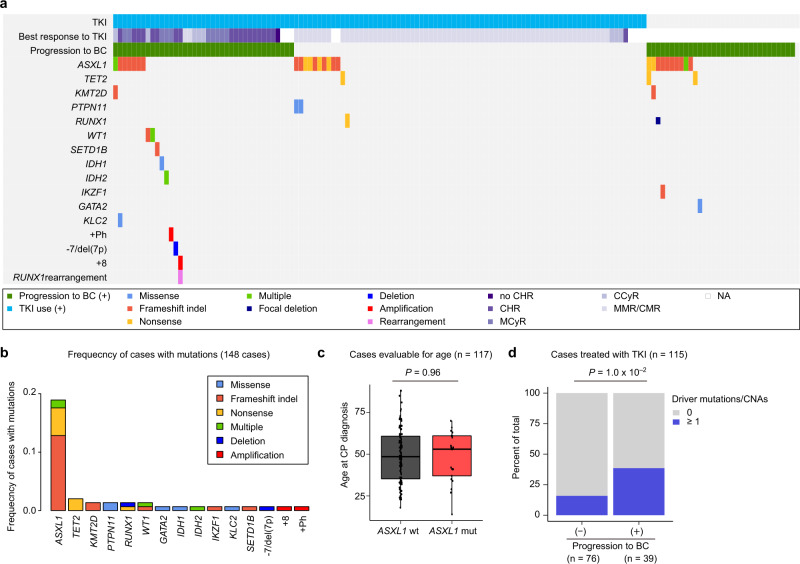
Genetic landscape of CML-CP. **a** Summary of genetic lesions in 148 CP patients. Each column indicates one patient. Clinical annotations including TKI usage for CP treatment, best response to TKI, and progression to BC are also shown. NA not available. **b** Frequencies of mutations in 148 CP patients. Recurrently mutated or known driver genes are described. Categories of mutations are depicted in different colours, and “multiple” indicates ≥2 distinct mutations found in the same gene in the same patient. **c** Box plot showing age at CP diagnosis according to the presence of *ASXL1* mutations. The median, first, and third quartiles are indicated and whiskers extend to the furthest value within 1.5× the interquartile range. The two-sided Wilcoxon rank-sum test was used to calculate the *P* value. wt wild type, mut mutated. **d** Proportion of cases harbouring any genetic abnormalities according to progression to BC. *P* values were evaluated using the Fisher’s exact test.

Finally, we explored the effect of genetic abnormalities on the clinical outcomes of CML-CP patients. Our cohort contained more patients who ultimately developed BC (48%, 71/148) compared to other cohorts, because we intentionally included paired CP and BC samples to investigate the molecular pathogenesis of the clonal evolution in CML (Fig. 1, Fig. 6a, and Supplementary Table 3). The median age at diagnosis for patients with CP was 49 (14–88) years and 77.7% of the patients had received TKI therapy, with imatinib being the most commonly used therapeutic agent (80%). Based on the best response, TKI induced complete haematologic response (CHR) in 17.9%, major/complete cytogenetic response (MCyR/CCyR) in 16.9%, and major/complete molecular response (MMR/CMR) in 64.2% of the patients.

In total, additional genetic alterations were found in 25.7% of the patients with CP at diagnosis. As expected based on the analysis described above (Fig. 1), only *ASXL1* was mutated in CP at a frequency comparable to that in BC, while other mutations, including *TET2*, *KMT2D*, *PTPN11*, *RUNX1*, and *WT1*, were found at much lower frequencies compared to those in BC (Fig. 6a, b, Supplementary Table 6, and Supplementary Data 2). Since, only *ASXL1* mutations were frequently found in CP and are common in age-related clonal haematopoiesis^27^, we evaluated the correlation between *ASXL1* mutations and patient age. Consistent with previous reports^10,13,28^, many patients with *ASXL1* mutations were younger than 60 years at the time of diagnosis of CP, as opposed to those with age-related clonal haematopoiesis. Moreover, there was no significant impact of age on the frequency of *ASXL1* mutations in CML-CP patients (Fig. 6c). We also evaluated whether genetic alterations in CP could predict BC progression in patients treated with TKIs. Interestingly, patients who received TKI and later experienced BC progression often harboured at least one mutation and/or CNA (38.5%, 15/24) than those without TKI treatment (15.8%, 12/76; *P* = 1.0 × 10^−2^; Fig. 6d). Thus, even when rarely observed, genetic alterations in CP play a role in driving CML cells to undergo transformation to BC under TKI pressure, which is in agreement with previous reports^10,29,30^.

## Discussion

Based on a comprehensive genetic analysis of a large cohort of CML patients with detailed clinical information and longitudinal sampling, we demonstrated the genetic landscape, clonal evolution, and their relevance to clinical features and outcomes in CML-CP/BC. Although the development of TKIs has substantially improved the outcomes of patients with CML-BC, the prognosis of BC patients remains bleak. To improve the clinical outcome of BC patients, it may be useful to stratify patients according to reliable prognostic markers to enable better clinical decision-making. We demonstrated that certain genetic lesions were better predictors of survival than clinical parameters. For instance, patients with multiple-hit *TP53* mutations and i(17q) presented with a particularly dismal outcome, which was consistent with previous studies reporting poor outcomes in *TP53*-mutated patients with other myeloid neoplasms, such as acute myeloid leukaemia and myelodysplastic syndromes^19,25,31–33^, and in CML-BC patients with i(17q)^34–36^. Moreover, *ASXL1* mutations, complex CNAs, i(17q), and +21 were independent predictors of poor prognosis in patients receiving TKI-based therapy based on multivariate analysis. Despite the limited number of samples analysed, our results indicate that genetic abnormalities can be effectively used as biomarkers to predict the outcome of BC patients to guide clinical decisions. As the *ABL1* mutation itself was not associated with a worse prognosis of CML-BC and was almost always accompanied by other mutations, targeting other mutations in combination with TKIs may be a promising treatment strategy in the future. Of note, sensitivity to various drugs is associated with the mutational profile of patients with acute myeloid leukaemia^37^, raising the possibility that in the near future, treatment may be personalised depending on individual mutational profiles.

Exome analysis of serial samples revealed that CML cells accumulated somatic mutations over time, which was markedly suppressed by TKI-based therapy. Although the precise reason for this remains unclear, it is possible that the application of TKIs eliminated the rapidly cycling BCR-ABL1-harbouring cells, ignoring the slow-cycling cells, which are less likely to ameliorate the impact of somatic mutations. The TKI-mediated reduction in blastic transformation may be attributable to a drastic reduction in the number of tumour cells at a risk of acquiring driver mutations for BC. However, our results suggest that the suppression of random mutational events may contribute to the reduced risk of BC in TKI-treated patients.

As previously reported^3–17^, most CML-BC patients in our cohort acquired additional mutations, which not only included mutations previously reported in haematological malignancies and CML-BC, but also included previously unreported recurrent mutations, such as *NBEAL2, PHIP*, and *KLC2* mutations. *NBEAL2* encodes a protein with a role in megakaryocyte alpha-granule biogenesis and has been reported to be mutated in patients with Grey platelet syndrome^38–40^ and factor XIII deficiency^41^. In contrast, to the best of our knowledge, mutations in *PHIP* and *KLC2* have not been reported in the context of haematological disorders. We also identified a *RUNX1-ETS2* fusion. *RUNX1* rearrangements are well-known driver events, which were first identified in core-binding factor leukaemia (*RUNX1*-*RUNX1T1)* and are present in a variety of myeloid and lymphoid malignancies, which are represented by *RUNX1-MECOM* in CML-BC, *ETV6-RUNX1* in B-precursor acute lymphoblastic leukaemia, and other fusions with unknown partners^42^. The fusion discovered in our cohort is another *RUNX1* rearrangement involving *ETS2*, which encodes a key haematopoietic transcription factor. Considering that genetic alterations in *RUNX1* are not restricted to SNVs, but also appear as CNAs and SVs, application of NGS-based techniques to detect multiple variant types may be important to monitor cases in CML patients. Among the mutations detected in CML, *ASXL1* mutations are more likely to be present at the time of CP diagnosis, and showed comparable TCFs between CP and BC samples. Thus, *ASXL1* mutations are unique compared to other mutations, as they were rarely detected in CP samples, but expanded with the onset of BC. Since, patients with CP who subsequently developed BC were more likely to harbour mutations, such as *ASXL1* mutations, compared to those who did not harbour such mutations, and because BC patients with *ASXL1* mutations had a poor prognosis, CP patients harbouring *ASXL1* mutations may require careful management. As most patients received imatinib as the first-line TKI for CP in our cohort, second- or third-generation TKIs may be better candidates for therapy to improve the outcomes of these patients, as suggested by a recent study^29^. As expected, myeloid and lymphoid BC cases exhibited distinct molecular profiles, which was in agreement with a previous report that patients with CML-BC presented with distinct additional chromosomal alterations depending on the lineage of BC^11^. The genetic profile of CML-BC is also influenced by a prior history of TKI therapy before BC diagnosis, which may explain, at least in part, the differences in the genetic profiles of BC patients. In summary, our study demonstrates the diverse mutational profiles and clonal evolution of CML in a large cohort and bridges genetic abnormalities and clinical features, including outcomes. Our results will hopefully lead to the development of efficacious therapy and strategies for better management of patients with CML.

## Methods

### Patient samples

We performed WES, targeted capture sequencing, and/or deep amplicon sequencing, using 112 CML-BC and 71 CP samples at diagnosis from 130 patients at ten institutions enroled in this study, according to the protocols approved by the Institutional Review Boards (Fig. 1a and Supplementary Table 1). This study was approved by the institutional ethics committees of Kyoto University, Kobe City Medical Center General Hospital, Tokyo Medical University, Akita University Graduate School of Medicine, Juntendo University School of Medicine, Gifu University Hospital, Kurashiki Central Hospital, Hyogo College of Medicine, Dokkyo Medical University, the University of Tokyo (Japan), and Chang Gung Memorial Hospital-Linkou (Taiwan), and was performed in accordance with the Declaration of Helsinki. Informed consent was obtained from all participants. Clinical information from these patients was collected if available, and cases of 99 patients with CML-BC were assessed for survival. Combined with the external WES data of 24 BC^12,13^ and 77 CP^13,21,22^ patients, we comprehensively analysed a total of 136 BC and 148 CP samples obtained from 216 CML patients for SNVs, CNAs, and SVs (Fig. 1a and Supplementary Table 1). Peripheral blood or bone marrow samples and matched buccal samples (if available) were collected from the patients who participated in this study. Genomic DNA from the samples was extracted using the QIAamp DNA mini kit (Qiagen) or Genetra PureGene kit (Qiagen). RNA from the samples was extracted using Trizol (Invitrogen, Life Technologies Corporation, Carlsbad, CA, USA).

### Detection and quantification of fusion transcripts

RT-PCR for detection of *BCR-ABL1* fusion transcripts^43^ and RT-qPCR with TaqMan assay for *BCR-ABL1* (ref. ^44^) was conducted with our lab-specific conversion factor obtained from the international reference laboratory at Adelaide, Australia for International Scale (IS) calculation. *BCR*-*ABL1* levels were expressed as IS^45^, along with a log reduction value. SYBR-green RT-qPCR was performed using the ABI 7900HT system (Applied Biosystems, Foster City, CA) for *RUNX1-ETS2* determination using freshly frozen samples, identical to those used for quantification of the *BCR-ABL1* transcript, according to the manufacturer’s instructions. The primer set for the measurement of the *RUNX1-ETS2* transcript comprised RUNX1-ETS2-QF (5′-CTTCACAAACCCACCGCAAG-3′) and RUNX1-ETS2-QR (5′-AGGGAGTCTGAGCTCTCGAAG-3′) with the same *ABL1* internal control gene for *BCR-ABL1*. Each RT-qPCR reaction was performed in duplicate and the relative expression after analysis of follow-up samples was compared with that of the diagnostic sample, using comparative quantification (2−ΔΔCt method) and expressed as log reduction.

### Whole-exome sequencing

WES was performed using the SureSelect Human All Exon kit V6 (Agilent Technology, Santa Clara, CA, USA), according to the manufacturer’s instructions. Captured targets were sequenced using the HiSeq 2500, NovaSeq 6000 (Illumina), or DNBSEQ G400 (MGI) instrument with a standard 125- or 150-bp paired-end read protocol^46^. Sequence alignment and mutation calling were performed using the hg19 reference genome and Genomon pipeline (https://github.com/Genomon-Project). For analysis of recurrent mutations, data on mutations were called for (i) BC samples using either CP or germline samples (if available) as control, and (ii) CP samples using germline samples as controls (if available). Data on putative somatic mutations with (i) Fisher’s exact *P* value <0.05; (ii) *P* value for EBCall^47^ < 0.0001, were filtered by excluding (i) synonymous SNVs; (ii) variants occurring in repetitive genomic regions; and (iii) known single-nucleotide polymorphisms (SNPs) listed in the 1000 Genomes Project (October 2014 release), NCBI dbSNP build 138, National Heart, Lung, and Blood Institute (NHLBI) Exome Sequencing Project (ESP) 6500, or our in-house dataset. In addition, (i) pathogenic mutations found in >1 haematological malignancies in the COSMIC database (v84); (ii) mutations expected to cause premature termination of protein translation (frameshift or nonsense mutations), were reviewed using a less stringent filter with (i) Fisher’s exact *P* value <0.1; (ii) *P* value for EBCall < 0.01, and data were manually curated. Mapping errors were removed by performing visual inspection using the Integrative Genomics Viewer (IGV). For conducting paired analysis of CP and BC samples, data on the putative recurrent mutations were subjected to validation using deep amplicon sequencing in both CP and BC samples, and mutations with VAF ≥ 0.02 were considered to be validated. Primers used for deep amplicon sequencing are summarised in Supplementary Table 7. All variants validated by deep amplicon sequencing were found to possess VAF > 0.03 in the WES data. For cases without germline controls, we also independently analysed the WES data of the CP and BC samples without using controls, and filtered data on the candidate mutations using the criteria used for performing targeted capture sequencing, as described below to rescue potentially overlooked recurrent mutations already present at the time of CP diagnosis. By doing so, we created three mutation lists for cases analysed for both CP and BC without germline controls, i.e., somatic mutations acquired in BC compared to CP, and independently called candidate recurrent mutations in CP and BC. We then merged the mutation lists to generate a single list for each BC sample. To analyse the number of SNVs acquired during disease progression (Fig. 1b and Supplementary Fig. 1b, d), data on the mutations were called for BC samples using CP as control and data on somatic mutations with (i) Fisher’s exact *P* value <0.01; (ii) VAF > 0.05; (iii) *P* value for EBCall < 0.001, were filtered by excluding (i) variants occurring in repetitive genomic regions; (ii) SNPs listed in the database as described above. For the external cohort^13^, WES data of both CP and BC samples were available for 15 patients. Of these, 13 were subjected to analysis after excluding 1 case, which lacked information on progression time from CP to BC and 1 in which a much lower depth was observed compared to the other samples. The estimated TCFs harbouring the relevant mutation were calculated with the total copy number (TCN) of the region and observed VAF values as follows^19^; TCF = TCN × VAF for deletions, TCF = 2VAF for regions without copy-number changes, and TCF = TCN × VAF for gains.

### Targeted capture sequencing

Targeted capture sequencing was performed using the SureSelect custom kit (Agilent Technologies), for which 104 genes were selected from those found to be mutated in the WES data of 52 CML-CP and BC pairs, and/or known oncogenes or tumour suppressor genes in haematological malignancies. Sequencing, alignment, and mutation calling were performed as per the WES analysis, except for the filtering criteria. Relevant somatic mutation data with (i) VAF > 0.05; (ii) depth > 100; (iii) *P* value for EBCall < 0.0001, were filtered by exclusion based on (i) synonymous SNVs; (ii) variants present only in unidirectional reads; (iii) variants occurring in repetitive genomic regions; (iv) missense SNVs with VAF of 0.4–0.6 or <0.04; and (v) known variants listed in SNP databases (as described in the “Whole-exome sequencing” section). In addition, (i) pathogenic mutations found in >1 haematological malignancies in the COSMIC database (v84) and (ii) mutations expected to cause premature termination of protein translation were reviewed by using a less stringent filter with (i) VAF > 0.02; (ii) depth >50; (iii) *P* value for EBCall > 0.001, and data were manually curated.

### CNA and SV analysis

CNAs and SVs were detected using the CNACS algorithm and Genomon-SV pipeline, respectively^19,20^. Briefly, CNACS analyses sequencing depth and allele frequencies of heterozygous SNPs to determine genome-wide copy numbers and detect CNAs^19^. We also used the ExomeDepth package in R^48^ to detect microdeletion events occurring in the exons of the *IKZF1* and *RUNX1* genes. CNA data were manually curated using IGV. The Genomon-SV pipeline detects SVs by utilising both breakpoint-containing junction read pairs and improperly aligned read pairs^20^. Data on putative SVs detected by using the Genomon pipeline were filtered by removing (i) those with Fisher’s exact *P* value >0.03 and (ii) those present in control normal samples, whose breakpoints were manually inspected using IGV. Since sequencing reads are enriched in gene exons and target genes in WES and targeted capture sequencing, respectively, detection of SV breakpoints is limited to regions close to those covered by gene baits. Nevertheless, a few SVs were detected even when they occurred in intronic regions. For instance, our pipeline could detect a *RUNX1*-*ETS2* fusion as the breakpoint was observed in the intronic region close to the *ETS2* gene exon (Supplementary Fig. 3a). Moreover, we were able to identify the *BCR-ABL1* fusion gene with the typical breakpoint, in the intronic region, through targeted capture sequencing and WES. Through targeted capture sequencing, we identified *BCR*-*ABL1* fusions in 51 out of 60 cases (85%); however, only 4 out of 76 cases (5.3%) were detected by WES. Therefore, the ability to detect SVs in our pipeline largely depended on the location of breakpoints and gene baits. Rearrangements of immunoglobulin and T-cell receptors were detected as microdeletion events involving loci observed in the WES analysis. Complex CNAs were defined as the presence of ≥3 abnormal CNAs detected by CNACS. We considered the co-amplification of 9q and 22q derived from +Ph abnormality as a single event. Hyperdiploidy and hypodiploidy were defined as two or more gains (≥48) and losses (≤44) of chromosomes assessed by CNACS, respectively, and data on these two aspects were removed from those on complex CNAs.

### Statistical analysis

Statistical analyses were performed using R (v3.5.0). Comparisons between groups were based on the two-sided Wilcoxon rank-sum test for continuous data and the Fisher’s exact test for categorical data. The correlation between the number of acquired mutations and time necessary for progression from CP to BC (Fig. 1b) was assessed by using the Poisson regression model and the glm function in R. Pairwise correlation of genetic legions and clinical factors (Supplementary Figs. 6 and 8) was assessed by the Fisher’s exact test with Benjamini–Hochberg correction^49^. Molecular responses for CML were defined as follows: haematologic remission, IS ≥ 10% for CHR, IS < 10% for MCyR, IS < 1% for CCyR, IS < 0.1% for MMR, and IS < 0.0032% for CMR. Survival analysis was performed for 99 patients with CML-BC for whom survival and treatment data for BC were available, and observations were censored at the last follow-up. The median follow-up was 3.2 years in surviving patients, and 24 (24.2%) patients were alive at the last follow-up. The Kaplan–Meier method was used to estimate the OS, and the differences in OS were assessed using the survival package in R. The effects of genetic lesions on OS were evaluated by using the Cox proportional hazards regression model, and data were adjusted for clinical factors that were significantly associated with OS in the univariate analysis. For blood counts, the following rounded median values were used as the threshold: 50,000 (×10^3^/uL) for WBC counts, 9.3 (g/dL) for haemoglobin levels (Hb), and 96,000 (×10^3^/uL) for platelet counts (PLT). Old age was defined as age of a patient ≥60 years, as per a previous study^18^. Multivariate analysis was performed for patients treated with TKI-based regimens (*n* = 59) by performing Cox proportional hazards regression modelling with a stepwise selection of variables using *P* value to exclude variables, in which genetic or clinical factors with a univariate Cox *P* value <0.10 were considered. To evaluate the validity of the established model, we also conducted bootstrapping 100 times to construct the test models with the factors subjected to the multivariate modelling using the rms package in R. Each model was assessed for validity by calculating the concordance statistic and frequency of each covariate included in the models. All *P* values were calculated using two-sided tests, and *P* < 0.05 was considered to be statistically significant.

### Reporting summary

Further information on research design is available in the Nature Research Reporting Summary linked to this article.

## Supplementary information

Supplementary Information

Description of Additional Supplementary Files

Supplementary Data 1-2

Reporting Summary

## Data Availability

The WES data in this study are deposited in the European Genome-phenome Archive under accession code EGAS00001005075. The data is available under restricted access, and access can be obtained by contacting S.O. (sogawa-tky@umin.ac.jp). The public WES data used in this study are available in the European Genome-phenome Archive under accession code EGAS00001003071, and the European Nucleotide Archive under accession code PRJEB20846 (Supplementary Table 8). The remaining data are available within the article, Supplementary Information, or available from the authors upon request.
