# Supplementary material for: Clonal evolution and clinical implications of genetic abnormalities in blastic transformation of chronic myeloid leukaemia
Source: Nat Commun. 2021 May 14;12:2833. doi: 10.1038/s41467-021-23097-w (PMC8121838; doi:10.1038/s41467-021-23097-w)
Supplement: Supplementary file 1 — Supplementary Information [file 41467_2021_23097_MOESM1_ESM.pdf]

Supplementary Information

Clonal evolution and clinical implications of genetic abnormalities in blastic transformation of chronic myeloid leukaemia

Yotaro Ochi, Kenichi Yoshida, Ying-Jung Huang, Ming-Chung Kuo, Yasuhito Nannya, Ko Sasaki, Kinuko Mitani, Noriko Hosoya, Nobuhiro Hiramoto, Takayuki Ishikawa, Susan Branford, Naranie Shanmuganathan, Kazuma Ohyashiki, Naoto Takahashi, Tomoiku Takaku, Shun Tsuchiya, Nobuhiro Kanemura, Nobuhiko Nakamura, Yasunori Ueda, Satoshi Yoshihara, Rabindranath Bera, Yusuke Shiozawa, Lanying Zhao, June Takeda, Yosaku Watatani, Rurika Okuda, Hideki Makishima, Yuichi Shiraishi, Kenichi Chiba, Hiroko Tanaka, Masashi Sanada, Akifumi Takaori-Kondo, Satoru Miyano, Seishi Ogawa, and Lee-Yung Shih

Supplementary Fig. 1

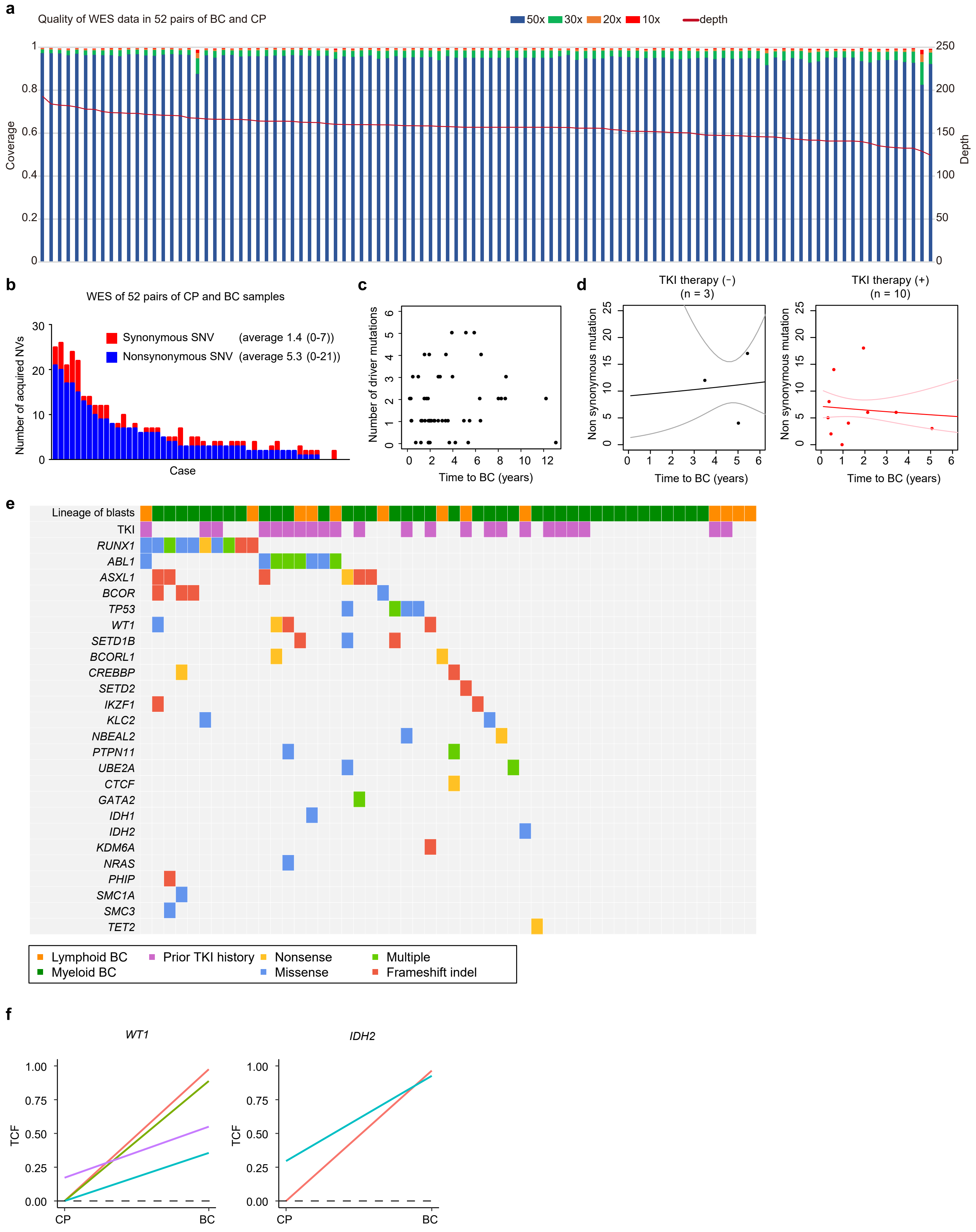

Supplementary Fig. 1. WES of CML-CP and BC samples. a, Sequencing quality of WES data in 52 pairs of CP and BC samples. Samples were sorted by mean depth. **b**, Number of acquired SNVs during evolution from CP to BC in 52 patients analysed for both CP and BC samples by WES. The average numbers and ranges are also described. **c**, Scatter plots showing time to progression to BC (horizontal axis) and number of recurrent mutations in CML-BC (vertical axis). **d**, Scatter plots for time to progression (horizontal axis) and number of acquired SNVs (vertical axis) during progression from CP to BC in 13 patients from an external cohort. Regression lines with 95% confidence intervals are also plotted. Cases with or without TKI therapy after CP diagnosis are indicated separately. **e**, Summary of mutations in CML-BC patients analysed by WES. Each column indicates one patient. Data on the lineage of blasts and prior history of TKI therapy before BC diagnosis are also shown. Categories of mutations are depicted in different colours, and “multiple” indicates ≥2 distinct mutations found in the same gene in the same patient. Mutations are ordered by frequency. **f**, TCFs of the indicated mutations in the corresponding CP and BC samples determined by deep amplicon sequencing. Black dashed lines indicate a TCF of 0%. Colours represent individual cases.

Supplementary Fig. 2

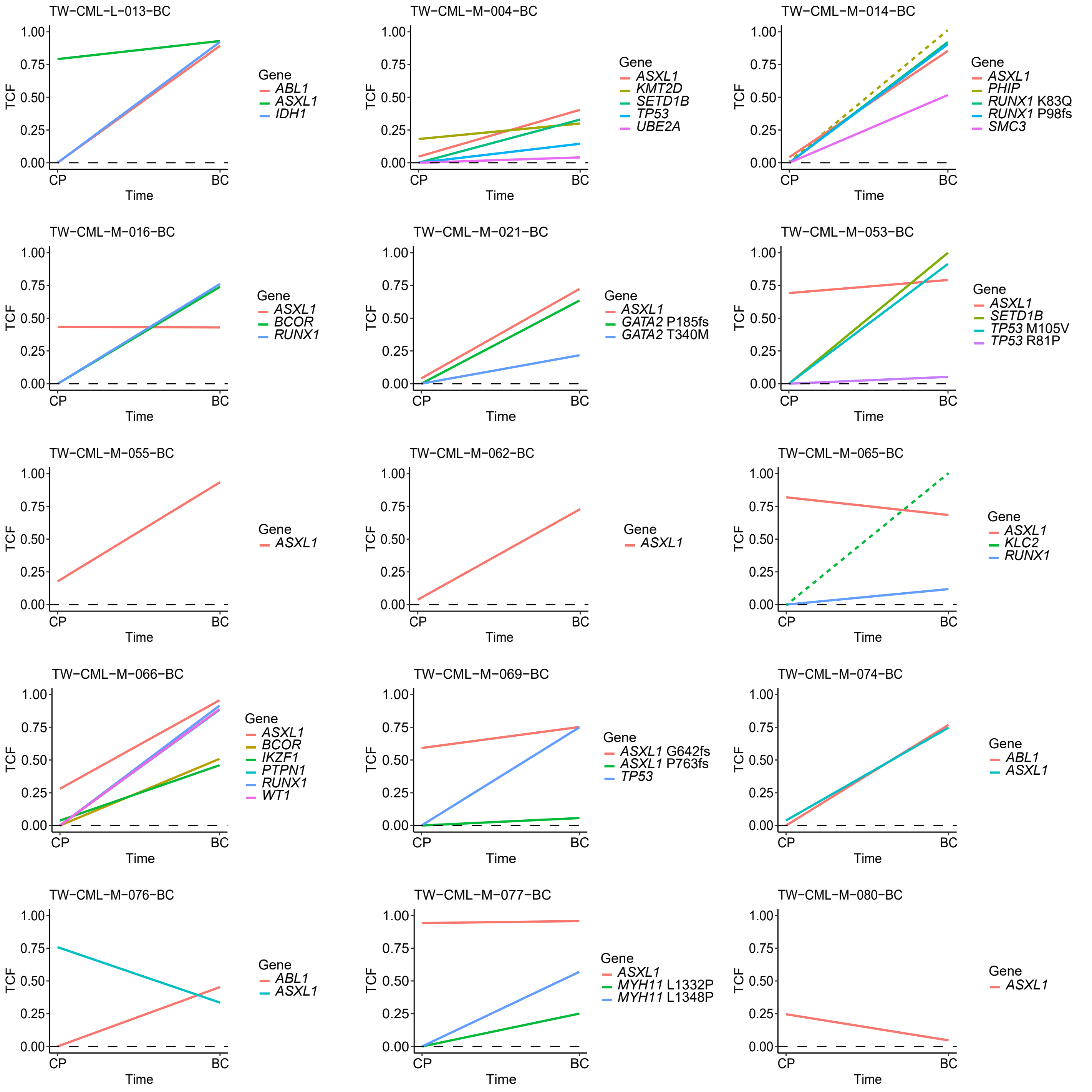

Supplementary Fig. 2. Relationship between *ASXL1* and other mutations during clonal evolution. TCFs of mutations determined by deep amplicon sequencing in the corresponding CP and BC samples of the indicated cases with *ASXL1* mutations. Black dashed lines indicate a TCF of 0%. Colours represent individual mutations. Mutations estimated to be >100% of TCFs are indicated as 100% in dashed lines. Note that two *MYH11* mutations (L1332P and L1348P) in a case (TW-CML-M-077) were found in different alleles, which was confirmed by IGV.

Supplementary Fig. 3

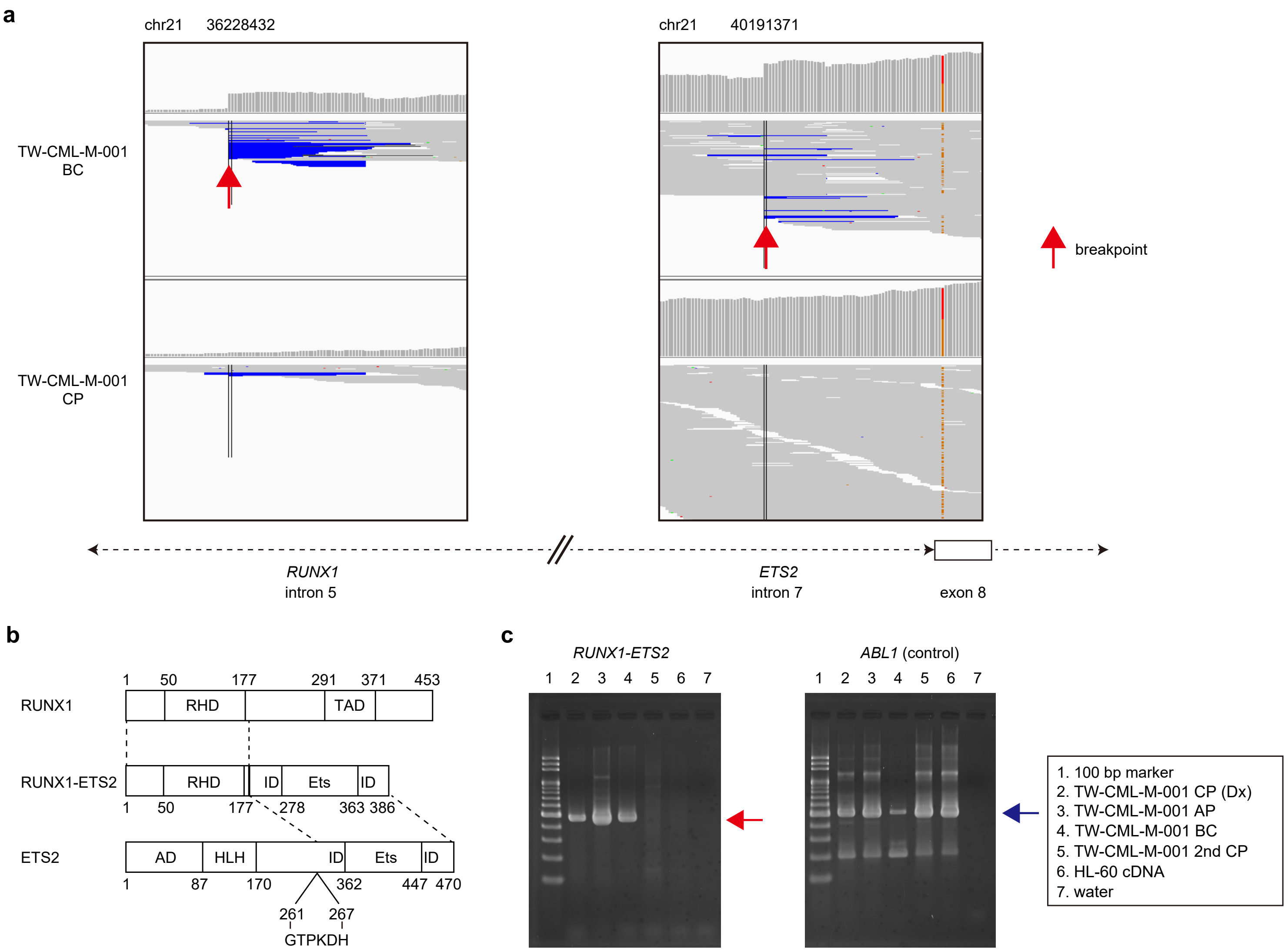

Supplementary Fig. 3. *RUNX1-ETS2* fusion in a patient with CML. **a**, Breakpoints of *RUNX1* and *ETS2* gene loci in a case with *RUNX1-ETS2* fusion (TW-CML-M-001), visualised by using the Integrated Genome Viewer (IGV). Although both breakpoints were within intronic regions, our pipeline could detect this SV as the breakpoint in the *ETS2* gene loci was close to the exon and could be captured by WES. **b**, Scheme depicting the expected in-frame fusion protein generated by the *RUNX1-ETS2* fusion. RHD, runt-homology domain; TAD, transactivation domain; AD, activation domain; HLH, helix-loop-helix domain; ID, inhibitory domain; Ets, Ets domain. **c**, RT-PCR of cDNA derived from a patient with *RUNX1-ETS2* fusion at diagnosis of CP, accelerated phase (AP), diagnosis of BC, and secondary CP. HL-60 cell line-derived cDNA and water were used as controls for the experiment. The expected band sizes of amplicons showing *RUNX1-ETS2* fusion and *ABL1* (as a control) are indicated by red and blue arrows, respectively.

Supplementary Fig. 4

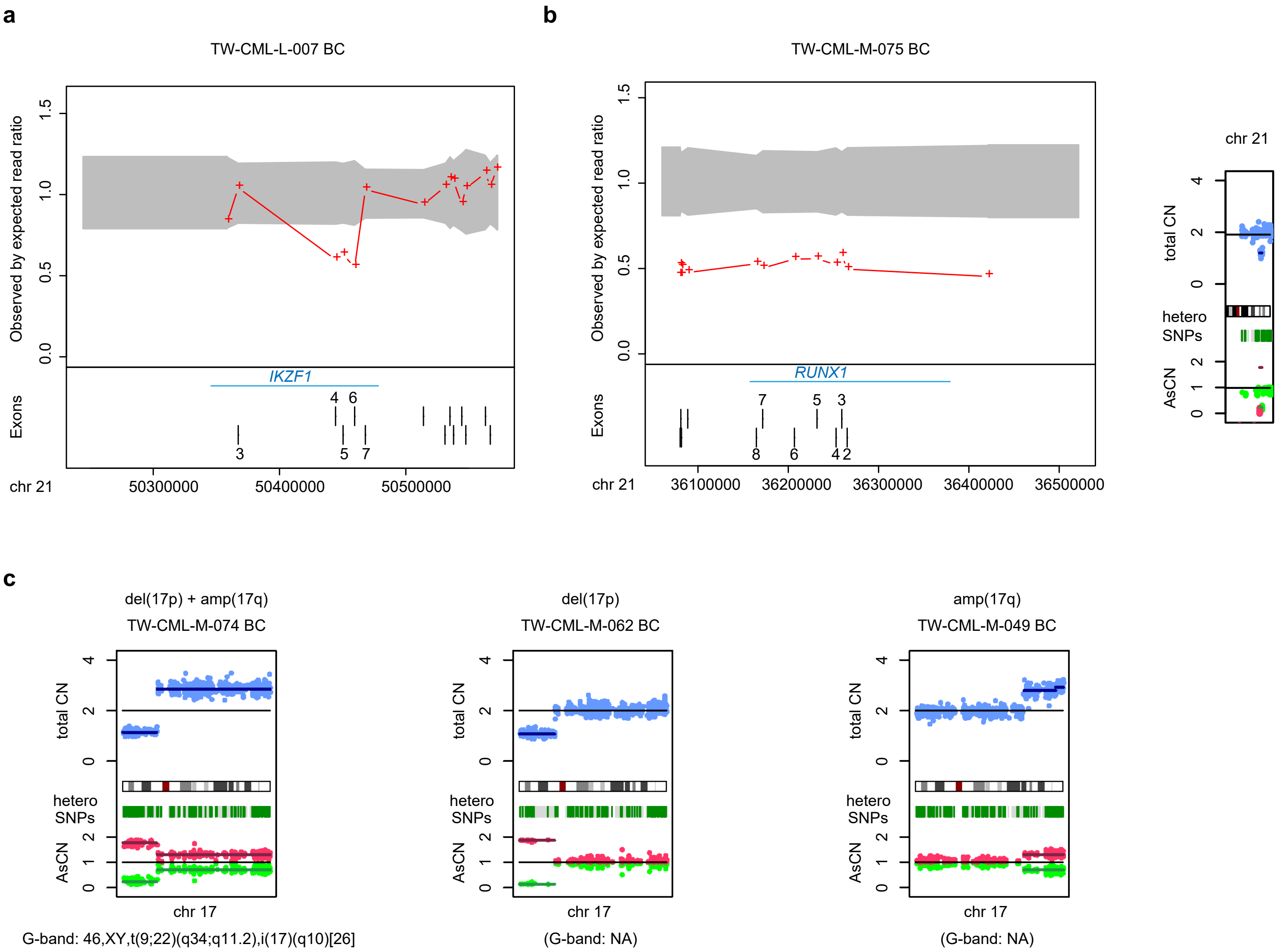

Supplementary Fig. 4. Detection of focal and chromosomal CNAs. **a**, Deletion in exons 4-6 of the *IKZF1* gene in a patient with CML-BC (TW-CML-L-007) evaluated by using the ExomeDepth package in R. The ratio between observed and expected read depth and genomic locus are shown in the vertical and horizontal axes, respectively. The 95% confidence interval is marked by using a grey shaded area. **b**, *RUNX1* gene deletion in a patient with CML-BC (TW-CML-M-075) detected by using the ExomeDepth (left) and CNACS algorithm (right). CN, copy-number; AsCN, allele-specific copy-number. **c**, Representative results of sequencing-based copy number profiling showing del(17p) and/or amp(17q). i(17q) is a known driver event in CML-BC, resulting in one copy of 17p and three copies of 17q, and can be successfully detected by conducting sequencing-based copy number profiling, as illustrated in the left panel. We also noticed that a few cases harboured either del(17p) or amp(17q), as represented in the cases illustrated in the middle and right panels. NA, not available.

Supplementary Fig. 5

a

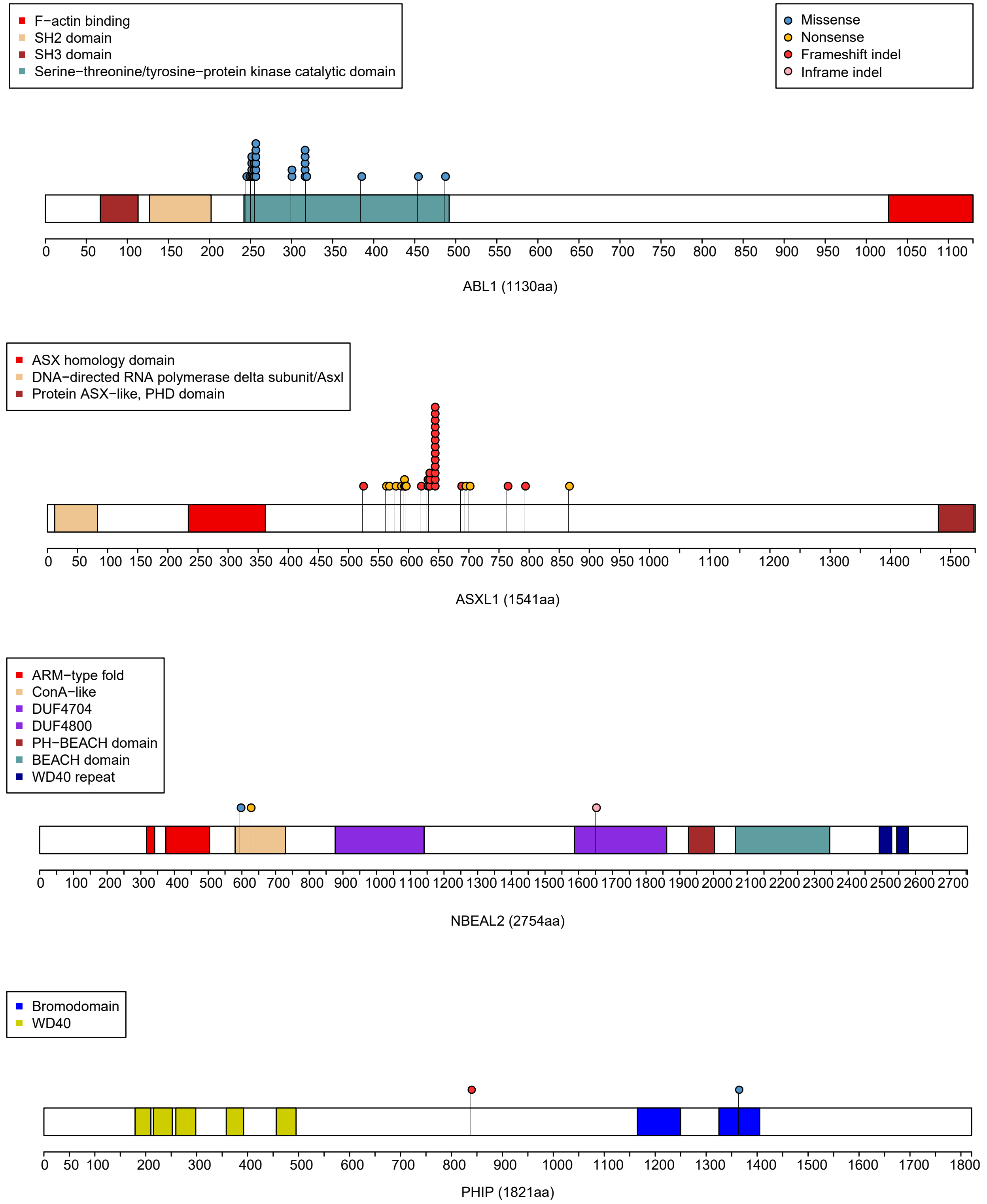

Supplementary Fig. 5. Distribution of mutations. Positions and types of mutations in the indicated proteins found in BC patients. Each circle and colour indicate the mutation and type of mutation, respectively. Functional domains are indicated in the coloured bands.

Supplementary Fig. 6

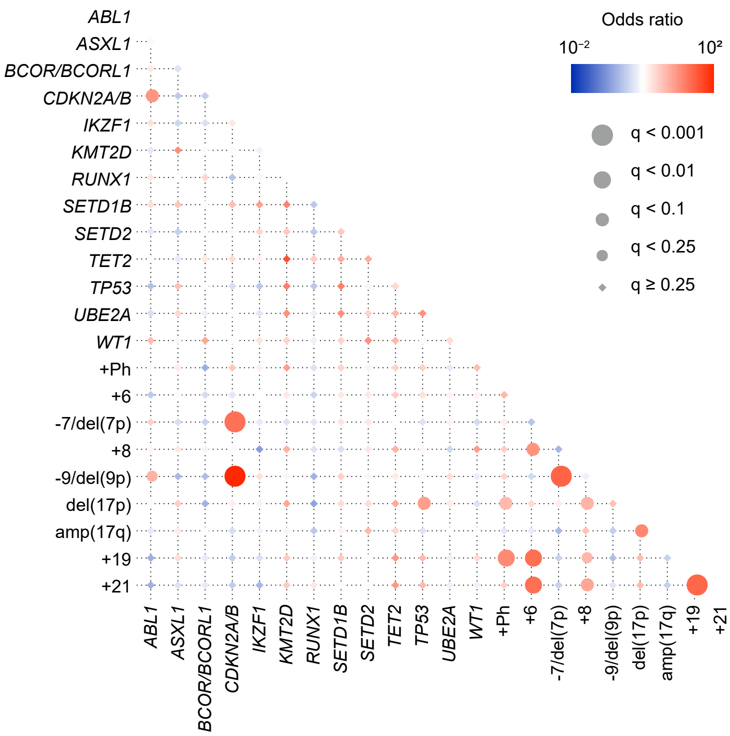

Supplementary Fig. 6. Correlation between genetic lesions in CML-BC. Correlations between genetic lesions in CML-BC. Co-occurring and mutually exclusive lesions are shown as red and blue circles, respectively. Odds ratio and the associated q-value are indicated by the colour gradient and the size of the circles, respectively.

Supplementary Fig. 7

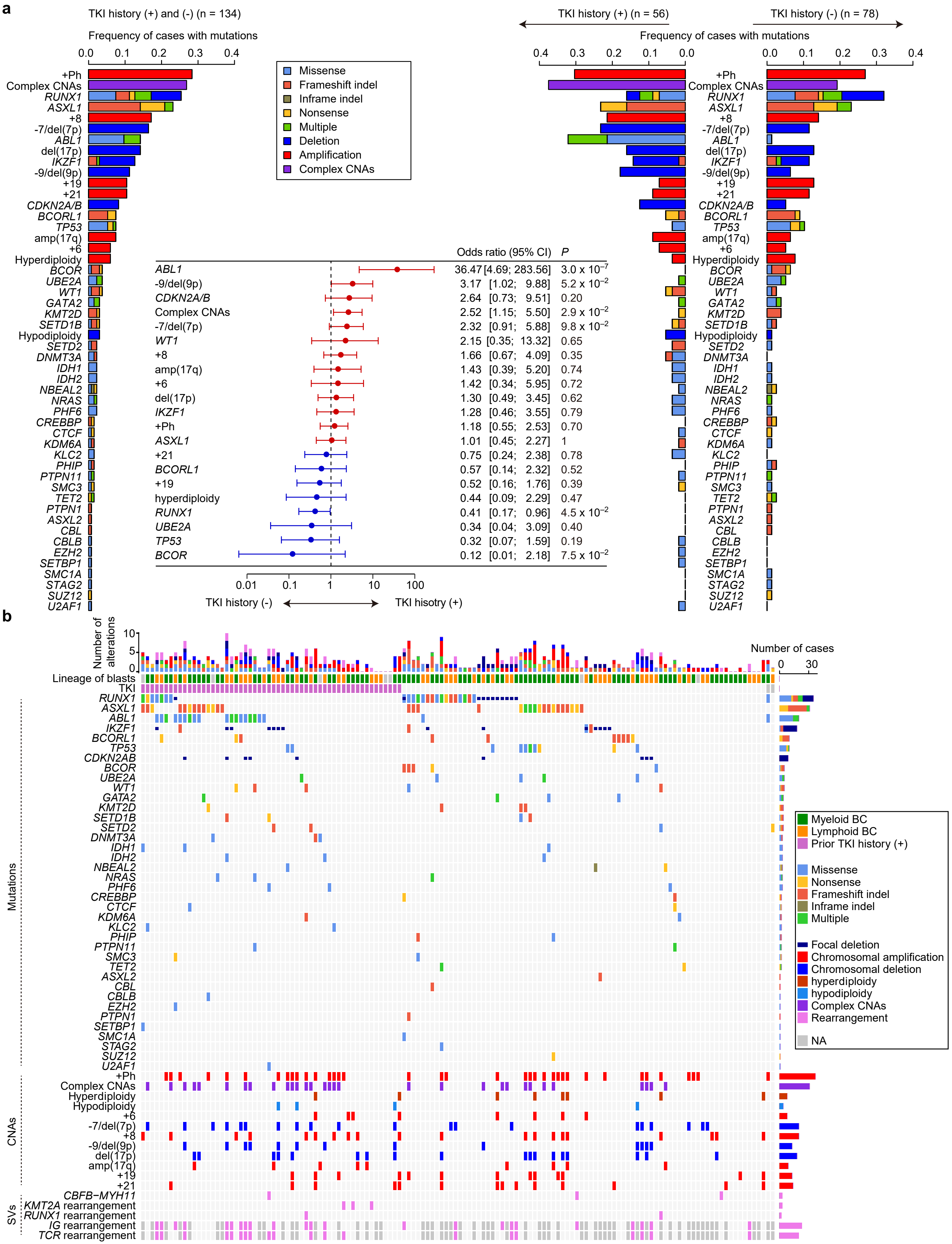

Supplementary Fig. 7. Genetic landscape of CML-BC with or without prior history of TKI therapy. **a**, Frequencies of mutations in 134 BC patients evaluable for prior history of TKI therapy (left panel), and those with (n = 56) or without prior TKI treatment history (n = 78) (right panel). Categories of mutations are depicted in different colours, and "multiple" indicates ≥2 distinct mutations found in the same gene in the same patient. The forest plot at the bottom shows odds ratios with 95% CIs for enrichment of each genetic lesion in cases with a prior history of TKI therapy. The dashed line represents an odds ratio of 1. Positive and negative odds ratios are indicated by red and blue colours, respectively. Genetic lesions found in >4 cases were included. *P*-values were calculated using the Fisher's exact test. **b,** Summary of genetic lesions in BC patients with or without prior history of TKI therapy. Each column indicates one patient. Types of alterations are depicted in different colours, and "multiple" indicates ≥2 distinct mutations found in the same gene in the same patient. The rearrangement of immunoglobulin (*IG*) and T-cell receptor (*TCR*) genes is shown in cases analysed by WES. NA, not available.

Supplementary Fig. 8

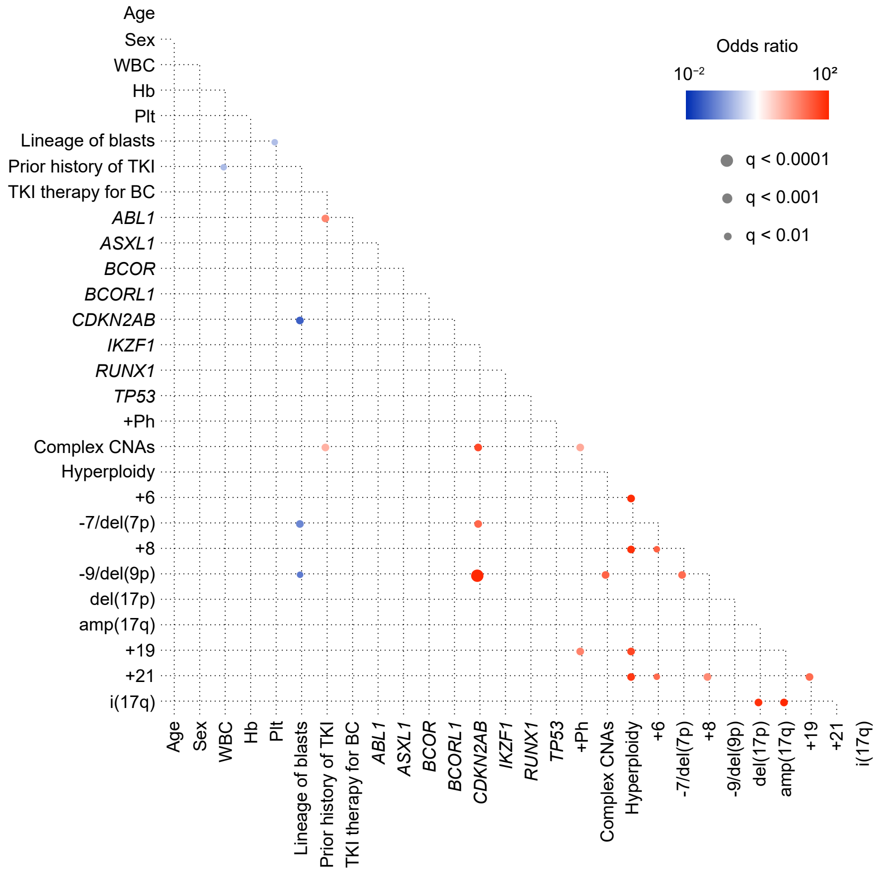

Supplementary Fig. 8. Correlation of genetic and clinical factors in survival analysis of CML-BC. Correlations between genetic/clinical factors in survival analysis of 99 BC patients. Co-occurring and mutually exclusive factors are shown in red and blue circles, respectively. Odds ratio and the associated q-value are indicated by the colour gradient and the size of the circles, respectively.

Supplementary Fig. 9

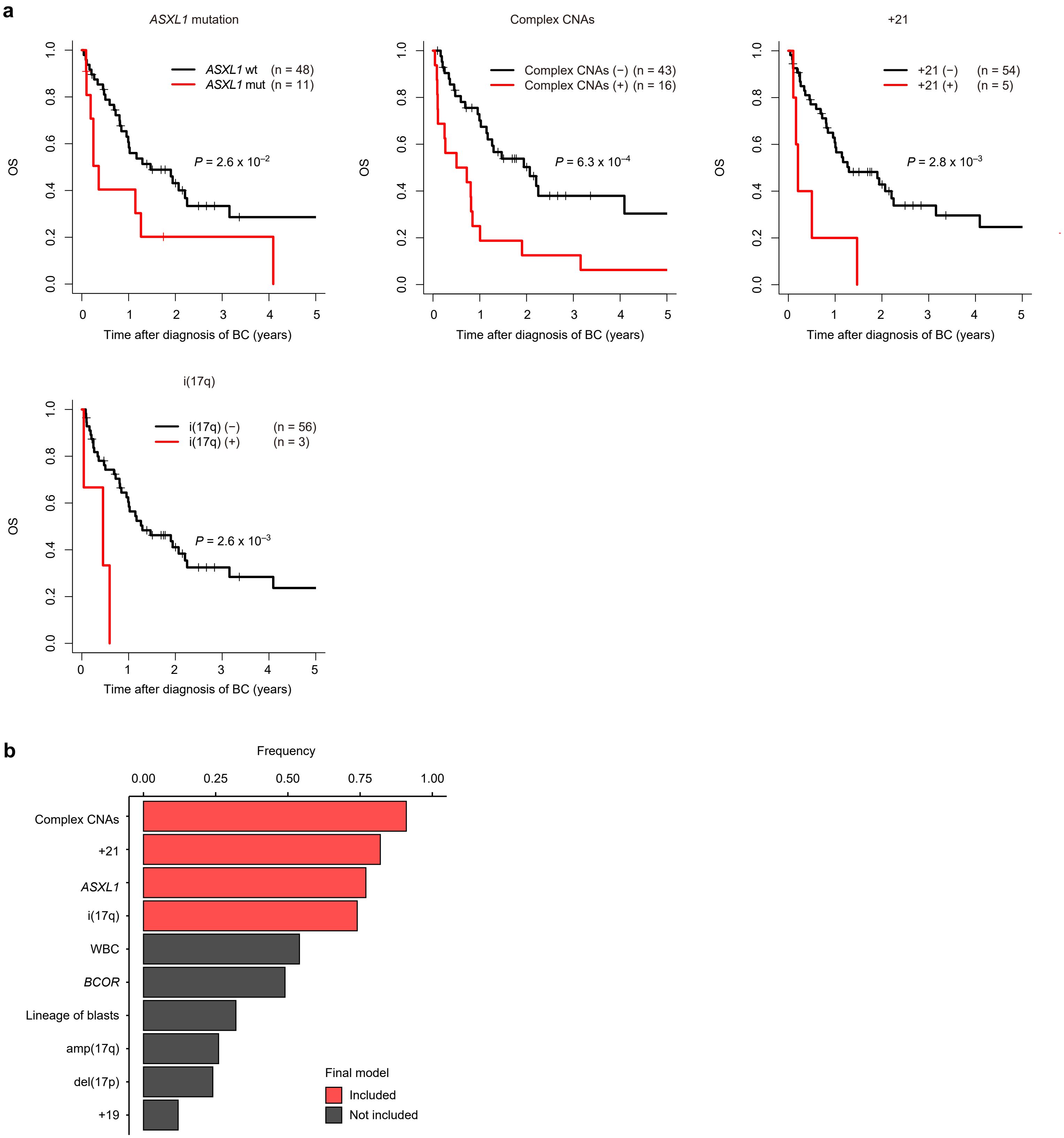

Supplementary Fig. 9. Prognostic impact of genetic abnormalities in patients with CML-BC treated with TKI-based therapy. a, Kaplan-Meier survival curves for OS in 59 patients with BC who were treated with TKI-based therapy according to the indicated clinical or genetic factors. The prognostic impact of each factor on OS was calculated using the log-rank test. **b**, Frequencies of each variable included in 100 bootstrapping and Cox proportional hazards regression modelling with a stepwise variable selection. The variables included in the final model, shown in Fig. 5b, are indicated in red.

Supplementary Fig. 10

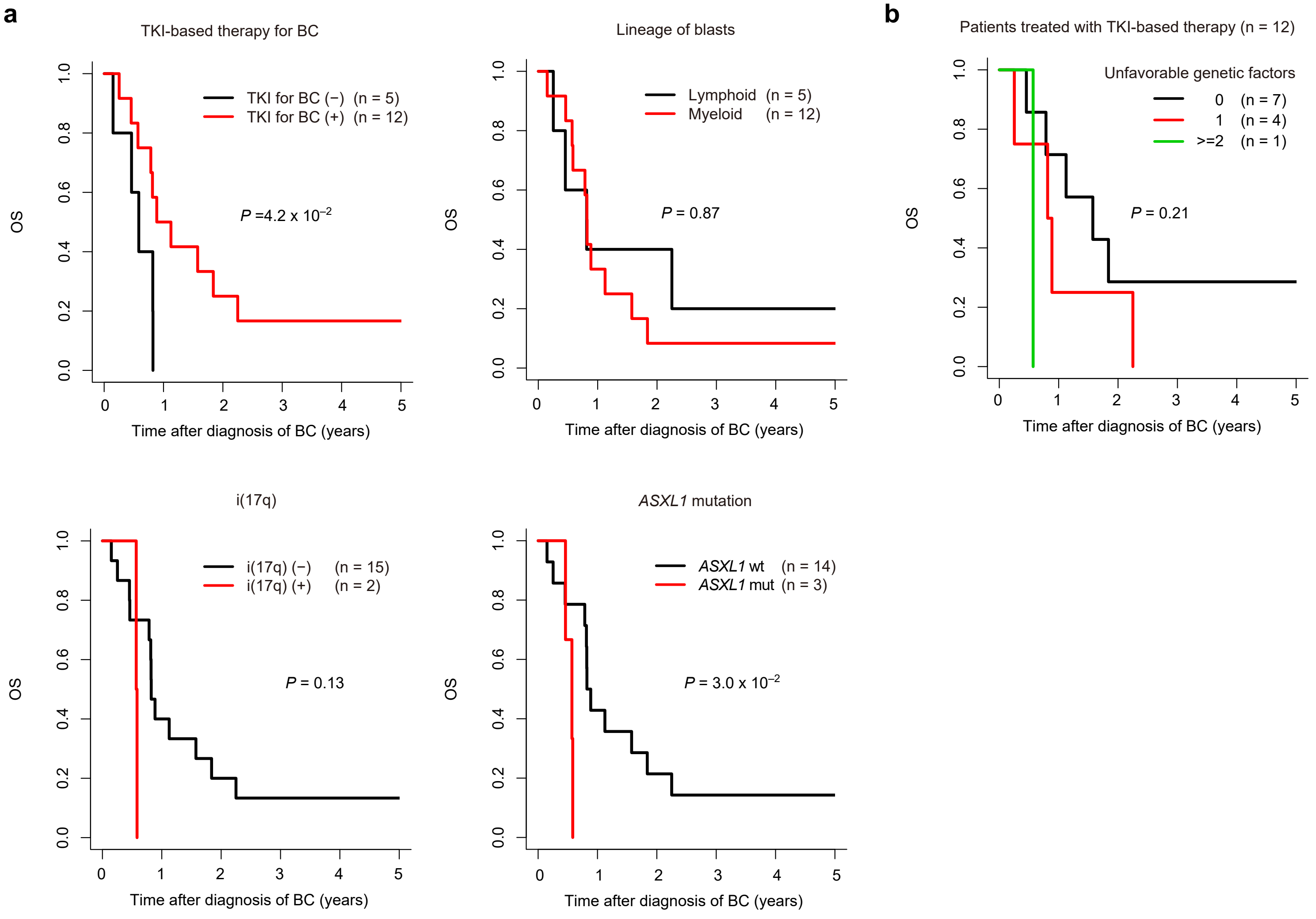

Supplementary Fig. 10. Prognostic impact of genetic abnormalities in patients with CML-BC in the external cohort. a, Kaplan-Meier survival curves for OS in 17 patients with BC in the external cohort according to the indicated clinical or genetic factors. The prognostic impact of each factor on OS was calculated using the log-rank test. **b**, Kaplan-Meier survival curves for OS in 12 patients who were treated with TKI-based therapy according to the number of genetic risk factors listed in Fig. 5b. *P*-values were calculated using the log-rank test.

Supplementary Table 1: Summary of analysed cases

samplename-BC	samplename-CP	all	WES-BC	WES-CP	WES-germ	target-BC	target-CP	inhouse_BC	inhouse_CP	published	publication	institution
CML_BC1		1	0	0	0	1	0	1	0	0		The University of Tokyo
CML_BC16		1	0	0	0	1	0	1	0	0		The University of Tokyo
CML_BC17		1	0	0	0	1	0	1	0	0		The University of Tokyo
CML_BC18		1	0	0	0	1	0	1	0	0		The University of Tokyo
CML_BC2		1	0	0	0	1	0	1	0	0		The University of Tokyo
CML_BC20		1	0	0	0	1	0	1	0	0		The University of Tokyo
CML_BC21		1	0	0	0	1	0	1	0	0		The University of Tokyo
CML_BC22		1	0	0	0	1	0	1	0	0		The University of Tokyo
CML_BC23		1	0	0	0	1	0	1	0	0		The University of Tokyo
CML_BC24		1	0	0	0	1	0	1	0	0		The University of Tokyo
CML_BC25		1	0	0	0	1	0	1	0	0		The University of Tokyo
CML_BC3		1	0	0	0	1	0	1	0	0		The University of Tokyo
CML_BC4		1	0	0	0	1	0	1	0	0		The University of Tokyo
CML_BC8		1	0	0	0	1	0	1	0	0		The University of Tokyo
CML_BC9		1	0	0	0	1	0	1	0	0		The University of Tokyo
DU_CML_BC002	DU_CML_BC001	1	0	0	0	1	1	1	1	0		Dokkyo Medical University
DU_CML_BC003		1	0	0	0	1	0	1	0	0		Dokkyo Medical University
DU_CML_BC004		1	0	0	0	1	0	1	0	0		Dokkyo Medical University
DU_CML_BC005		1	0	0	0	1	0	1	0	0		Dokkyo Medical University
DU_CML_BC006		1	0	0	0	1	0	1	0	0		Dokkyo Medical University
DU_CML_BC007		1	0	0	0	1	0	1	0	0		Dokkyo Medical University
DU_CML_BC008		1	0	0	0	1	0	1	0	0		Dokkyo Medical University
DU_CML_BC009		1	0	0	0	1	0	1	0	0		Dokkyo Medical University
	DU_CML_BC010	1	0	0	0	0	1	0	1	0		Dokkyo Medical University
	DU_CML_BC011	1	0	0	0	0	1	0	1	0		Dokkyo Medical University
	DU_CML_BC012	1	0	0	0	0	1	0	1	0		Dokkyo Medical University
	DU_CML_BC013	1	0	0	0	0	1	0	1	0		Dokkyo Medical University
	DU_CML_BC014	1	0	0	0	0	1	0	1	0		Dokkyo Medical University
	DU_CML_BC015	1	0	0	0	0	1	0	1	0		Dokkyo Medical University
	DU_CML_BC016	1	0	0	0	0	1	0	1	0		Dokkyo Medical University
	DU_CML_BC017	1	0	0	0	0	1	0	1	0		Dokkyo Medical University
	DU_CML_BC018	1	0	0	0	0	1	0	1	0		Dokkyo Medical University
	DU_CML_BC019	1	0	0	0	0	1	0	1	0		Dokkyo Medical University
	DU_CML_BC020	1	0	0	0	0	1	0	1	0		Dokkyo Medical University
	DU_CML_BC021	1	0	0	0	0	1	0	1	0		Dokkyo Medical University
	DU_CML_BC022	1	0	0	0	0	1	0	1	0		Dokkyo Medical University
	DU_CML_BC023	1	0	0	0	0	1	0	1	0		Dokkyo Medical University
	DU_CML_BC024	1	0	0	0	0	1	0	1	0		Dokkyo Medical University
	DU_CML_BC025	1	0	0	0	0	1	0	1	0		Dokkyo Medical University
	DU_CML_BC026	1	0	0	0	0	1	0	1	0		Dokkyo Medical University
	DU_CML_BC027	1	0	0	0	0	1	0	1	0		Dokkyo Medical University
DU_CML_BC028		1	0	0	0	1	0	1	0	0		Dokkyo Medical University
KCGH_256_1		1	0	0	0	1	0	1	0	0		Kobe City Medical Center General Hospital
KCGH203-BC	KCGH203-D	1	1	1	1	0	0	1	1	0		Kobe City Medical Center General Hospital
TW-CML-L-001-BC	TW-CML-L-001-D	1	1	1	0	0	0	1	1	0		Chang Gung Memorial Hospital-Linkou
TW-CML-L-002-BC	TW-CML-L-002-D	1	1	1	0	0	0	1	1	0		Chang Gung Memorial Hospital-Linkou
TW-CML-L-003-BC	TW-CML-L-003-D	1	1	1	0	0	0	1	1	0		Chang Gung Memorial Hospital-Linkou
TW-CML-L-004-BC	TW-CML-L-004-D	1	1	1	0	0	0	1	1	0		Chang Gung Memorial Hospital-Linkou
TW-CML-L-005-BC	TW-CML-L-005-D	1	1	1	0	0	0	1	1	0		Chang Gung Memorial Hospital-Linkou
TW-CML-L-007-BC	TW-CML-L-007-D	1	1	1	0	0	0	1	1	0		Chang Gung Memorial Hospital-Linkou
TW-CML-L-009-BC	TW-CML-L-009-D	1	1	1	0	0	0	1	1	0		Chang Gung Memorial Hospital-Linkou
TW-CML-L-010-BC	TW-CML-L-010-D	1	1	1	0	0	0	1	1	0		Chang Gung Memorial Hospital-Linkou
TW-CML-L-011		1	0	0	0	1	0	1	0	0		Chang Gung Memorial Hospital-Linkou
TW-CML-L-012-BC	TW-CML-L-012-D	1	1	1	0	0	0	1	1	0		Chang Gung Memorial Hospital-Linkou
TW-CML-L-013-BC	TW-CML-L-013-D	1	1	1	0	0	0	1	1	0		Chang Gung Memorial Hospital-Linkou
TW-CML-L-014-BC	TW-CML-L-014-D	1	1	1	0	0	0	1	1	0		Chang Gung Memorial Hospital-Linkou
TW-CML-L-015-BC	TW-CML-L-015-D	1	1	1	0	0	0	1	1	0		Chang Gung Memorial Hospital-Linkou
TW-CML-M-001-BC	TW-CML-M-001-D	1	1	1	0	0	0	1	1	0		Chang Gung Memorial Hospital-Linkou
TW-CML-M-004-BC	TW-CML-M-004-D	1	1	1	0	0	0	1	1	0		Chang Gung Memorial Hospital-Linkou
TW-CML-M-006-BC	TW-CML-M-006-D	1	1	1	0	0	0	1	1	0		Chang Gung Memorial Hospital-Linkou
TW-CML-M-007-BC	TW-CML-M-007-D	1	1	1	0	0	0	1	1	0		Chang Gung Memorial Hospital-Linkou
TW-CML-M-010-BC	TW-CML-M-010-D	1	1	1	0	0	0	1	1	0		Chang Gung Memorial Hospital-Linkou
TW-CML-M-014-BC	TW-CML-M-014-D-2	1	1	1	0	0	0	1	1	0		Chang Gung Memorial Hospital-Linkou
TW-CML-M-016-BC	TW-CML-M-016-D	1	1	1	0	0	0	1	1	0		Chang Gung Memorial Hospital-Linkou
TW-CML-M-018-BC	TW-CML-M-018-D	1	1	1	0	0	0	1	1	0		Chang Gung Memorial Hospital-Linkou
TW-CML-M-021-BC	TW-CML-M-021-D	1	1	1	0	0	0	1	1	0		Chang Gung Memorial Hospital-Linkou
TW-CML-M-049-BC	TW-CML-M-049-D	1	1	1	0	0	0	1	1	0		Chang Gung Memorial Hospital-Linkou
TW-CML-M-050-BC	TW-CML-M-050-D	1	1	1	0	0	0	1	1	0		Chang Gung Memorial Hospital-Linkou
TW-CML-M-051-BC	TW-CML-M-051-D	1	1	1	0	0	0	1	1	0		Chang Gung Memorial Hospital-Linkou
TW-CML-M-052-BC	TW-CML-M-052-D	1	1	1	0	0	0	1	1	0		Chang Gung Memorial Hospital-Linkou
TW-CML-M-053-BC	TW-CML-M-053-D	1	1	1	0	0	0	1	1	0		Chang Gung Memorial Hospital-Linkou
TW-CML-M-054-BC	TW-CML-M-054-D	1	1	1	0	0	0	1	1	0		Chang Gung Memorial Hospital-Linkou
TW-CML-M-055-BC	TW-CML-M-055-D	1	1	1	0	0	0	1	1	0		Chang Gung Memorial Hospital-Linkou
TW-CML-M-056-BC	TW-CML-M-056-D	1	1	1	0	0	0	1	1	0		Chang Gung Memorial Hospital-Linkou
TW-CML-M-058		1	0	0	0	1	0	1	0	0		Chang Gung Memorial Hospital-Linkou
TW-CML-M-059-BC	TW-CML-M-059-D	1	1	1	0	0	0	1	1	0		Chang Gung Memorial Hospital-Linkou
TW-CML-M-060-BC	TW-CML-M-060-D	1	1	1	0	0	0	1	1	0		Chang Gung Memorial Hospital-Linkou
TW-CML-M-061-BC	TW-CML-M-061-D	1	1	1	0	0	0	1	1	0		Chang Gung Memorial Hospital-Linkou
TW-CML-M-062-BC	TW-CML-M-062-D	1	1	1	0	0	0	1	1	0		Chang Gung Memorial Hospital-Linkou
TW-CML-M-063-BC	TW-CML-M-063-D	1	1	1	0	0	0	1	1	0		Chang Gung Memorial Hospital-Linkou
TW-CML-M-064-BC	TW-CML-M-064-D	1	1	1	0	0	0	1	1	0		Chang Gung Memorial Hospital-Linkou
TW-CML-M-065-BC	TW-CML-M-065-D	1	1	1	0	0	0	1	1	0		Chang Gung Memorial Hospital-Linkou
TW-CML-M-066-BC	TW-CML-M-066-D	1	1	1	0	0	0	1	1	0		Chang Gung Memorial Hospital-Linkou
TW-CML-M-067-BC	TW-CML-M-067-D	1	1	1	0	0	0	1	1	0		Chang Gung Memorial Hospital-Linkou
TW-CML-M-068-BC	TW-CML-M-068-D	1	1	1	0	0	0	1	1	0		Chang Gung Memorial Hospital-Linkou
TW-CML-M-069-BC	TW-CML-M-069-D	1	1	1	0	0	0	1	1	0		Chang Gung Memorial Hospital-Linkou
TW-CML-M-070		1	0	0	0	1	0	1	0	0		Chang Gung Memorial Hospital-Linkou
TW-CML-M-071-BC	TW-CML-M-071-D	1	1	1	0	0	0	1	1	0		Chang Gung Memorial Hospital-Linkou
TW-CML-M-072		1	0	0	0	1	0	1	0	0		Chang Gung Memorial Hospital-Linkou
TW-CML-M-073-BC	TW-CML-M-073-D	1	1	1	0	0	0	1	1	0		Chang Gung Memorial Hospital-Linkou
TW-CML-M-074-BC	TW-CML-M-074-D	1	1	1	0	0	0	1	1	0		Chang Gung Memorial Hospital-Linkou
TW-CML-M-075-BC	TW-CML-M-075-D	1	1	1	0	0	0	1	1	0		Chang Gung Memorial Hospital-Linkou
TW-CML-M-076-BC	TW-CML-M-076-D	1	1	1	0	0	0	1	1	0		Chang Gung Memorial Hospital-Linkou
TW-CML-M-077-BC	TW-CML-M-077-D	1	1	1	0	0	0	1	1	0		Chang Gung Memorial Hospital-Linkou
TW-CML-M-078-BC	TW-CML-M-078-D	1	1	1	0	0	0	1	1	0		Chang Gung Memorial Hospital-Linkou
TW-CML-M-079-BC	TW-CML-M-079-D	1	1	1	0	0	0	1	1	0		Chang Gung Memorial Hospital-Linkou
TW-CML-M-080-BC	TW-CML-M-080-D	1	1	1	0	0	0	1	1	0		Chang Gung Memorial Hospital-Linkou
TW-CML-M-081		1	0	0	0	1	0	1	0	0		Chang Gung Memorial Hospital-Linkou
TW-CML-M-082-BC	TW-CML-M-082-D	1	1	1	0	0	0	1	1	0		Chang Gung Memorial Hospital-Linkou
TW-CML-M-083-BC	TW-CML-M-083-D	1	1	1	0	0	0	1	1	0		Chang Gung Memorial Hospital-Linkou
TW-CML-M-084		1	0	0	0	1	0	1	0	0		Chang Gung Memorial Hospital-Linkou
TW-CML-M-086		1	0	0	0	1	0	1	0	0		Chang Gung Memorial Hospital-Linkou
HCM-017-1		1	0	0	0	1	0	1	0	0		Hyogo College of Medicine
GIFU-143-1		1	0	0	0	1	0	1	0	0		Gifu University Hospital
GIFU-161-1		1	0	0	0	1	0	1	0	0		Gifu University Hospital
GIFU-239-1		1	0	0	0	1	0	1	0	0		Gifu University Hospital
GIFU-313-1		1	0	0	0	1	0	1	0	0		Gifu University Hospital
TMU-196-1		1	0	0	0	1	0	1	0	0		Tokyo Medical University
TMU-298-1		1	0	0	0	1	0	1	0	0		Tokyo Medical University
TMU-475-1		1	0	0	0	1	0	1	0	0		Tokyo Medical University
TMU-960-1		1	0	0	0	1	0	1	0	0		Tokyo Medical University
TMU-800-1		1	0	0	0	1	0	1	0	0		Tokyo Medical University
TMU-006-7		1	0	0	0	1	0	1	0	0		Tokyo Medical University
JUN-001		1	0	0	0	1	0	1	0	0		Juntendo University School of Medicine
JUN-002		1	0	0	0	1	0	1	0	0		Juntendo University School of Medicine
JUN-003		1	0	0	0	1	0	1	0	0		Juntendo University School of Medicine

JUN-004		1	0	0	0	1	0	1	0	0	Juntendo University School of Medicine
AKT-001		1	0	0	0	1	0	1	0	0	Akita University Graduate School of Medicine
AKT-003		1	0	0	0	1	0	1	0	0	Akita University Graduate School of Medicine
AKT-005		1	0	0	0	1	0	1	0	0	Akita University Graduate School of Medicine
AKT-006		1	0	0	0	1	0	1	0	0	Akita University Graduate School of Medicine
AKT-007		1	0	0	0	1	0	1	0	0	Akita University Graduate School of Medicine
KCGH-217-1		1	0	0	0	1	0	1	0	0	Kobe City Medical Center General Hospital
KCGH-350-1		1	0	0	0	1	0	1	0	0	Kobe City Medical Center General Hospital
KCGH-356-1		1	0	0	0	1	0	1	0	0	Kobe City Medical Center General Hospital
KCGH-421-1		1	0	0	0	1	0	1	0	0	Kobe City Medical Center General Hospital
KCGH-444-1		1	0	0	0	1	0	1	0	0	Kobe City Medical Center General Hospital
KCGH-210-1		1	0	0	0	1	0	1	0	0	Kobe City Medical Center General Hospital
KCGH-762-1		1	0	0	0	1	0	1	0	0	Kobe City Medical Center General Hospital
KCH-116-1		1	0	0	0	1	0	1	0	0	Kurashiki Central Hospital
1_MBC_Dx.WES		1	1	0	1	0	0	0	0	1	Branford et al. Blood 2018
10_LBC.WES	10_CP_Dx.WES	1	1	1	0	0	0	0	0	1	Branford et al. Blood 2018
	11_CP_Dx.WES	1	0	1	1	0	0	0	0	1	Branford et al. Blood 2018
12_LBC.WES	12_CP_Dx.WES	1	1	1	1	0	0	0	0	1	Branford et al. Blood 2018
13_LBC.WES	13_CP_Dx.WES	1	1	1	1	0	0	0	0	1	Branford et al. Blood 2018
14_LBC.WES	14_CP_Dx.WES	1	1	1	1	0	0	0	0	1	Branford et al. Blood 2018
15_MBC.WES	15_CP_Dx.WES	1	1	1	1	0	0	0	0	1	Branford et al. Blood 2018
16_LBC.WES	16_CP_Dx.WES	1	1	1	1	0	0	0	0	1	Branford et al. Blood 2018
17_MBC.WES	17_CP_Dx.WES	1	1	1	1	0	0	0	0	1	Branford et al. Blood 2018
18_MBC.WES	18_CP_Dx.WES	1	1	1	1	0	0	0	0	1	Branford et al. Blood 2018
19_MBC.WES	19_CP_Dx.WES	1	1	1	1	0	0	0	0	1	Branford et al. Blood 2018
2_MBC_Dx.WES		1	1	0	1	0	0	0	0	1	Branford et al. Blood 2018
20_MBC.WES		1	1	0	1	0	0	0	0	1	Branford et al. Blood 2018
21_MBC.WES	21_CP_Dx.WES	1	1	1	1	0	0	0	0	1	Branford et al. Blood 2018
22_MBC.WES		1	1	0	1	0	0	0	0	1	Branford et al. Blood 2018
23_MBC.WES	23_CP_Dx.WES	1	1	1	1	0	0	0	0	1	Branford et al. Blood 2018
	24_CP_Dx.WES	1	0	1	1	0	0	0	0	1	Branford et al. Blood 2018
	25_CP_Dx.WES	1	0	1	1	0	0	0	0	1	Branford et al. Blood 2018
	26_CP_Dx.WES	1	0	1	1	0	0	0	0	1	Branford et al. Blood 2018
	27_CP_Dx.WES	1	0	1	1	0	0	0	0	1	Branford et al. Blood 2018
	28_CP_Dx.WES	1	0	1	1	0	0	0	0	1	Branford et al. Blood 2018
	29_CP_Dx.WES	1	0	1	1	0	0	0	0	1	Branford et al. Blood 2018
3_MBC.WES		1	1	0	1	0	0	0	0	1	Branford et al. Blood 2018
	30_CP_Dx.WES	1	0	1	1	0	0	0	0	1	Branford et al. Blood 2018
	31_CP_Dx.WES	1	0	1	1	0	0	0	0	1	Branford et al. Blood 2018
	32_CP_Dx.WES	1	0	1	1	0	0	0	0	1	Branford et al. Blood 2018
	33_CP_Dx.WES	1	0	1	1	0	0	0	0	1	Branford et al. Blood 2018
	34_CP_Dx.WES	1	0	1	1	0	0	0	0	1	Branford et al. Blood 2018
	35_CP_Dx.WES	1	0	1	1	0	0	0	0	1	Branford et al. Blood 2018
	36_CP_Dx.WES	1	0	1	1	0	0	0	0	1	Branford et al. Blood 2018
	37_CP_Dx.WES	1	0	1	1	0	0	0	0	1	Branford et al. Blood 2018
4_LBC.WES	4_CP_Dx.WES	1	1	1	1	0	0	0	0	1	Branford et al. Blood 2018
	5_CP_Dx.WES	1	0	1	1	0	0	0	0	1	Branford et al. Blood 2018
	51_CP.WES	1	0	1	1	0	0	0	0	1	Branford et al. Blood 2018
6_MBC.WES	6_CP_Dx.WES	1	1	1	1	0	0	0	0	1	Branford et al. Blood 2018
7_LBC.WES	7_CP_Dx.WES	1	1	1	0	0	0	0	0	1	Branford et al. Blood 2018
	8_CP_Dx.WES	1	0	1	1	0	0	0	0	1	Branford et al. Blood 2018
9_LBC.WES	9_CP_Dx.WES	1	1	1	1	0	0	0	0	1	Branford et al. Blood 2018
	CML212_103_CP	1	0	1	1	0	0	0	0	1	Togasaki et al. Blood Cancer J 2017
	CML212_104_CP	1	0	1	1	0	0	0	0	1	Togasaki et al. Blood Cancer J 2017
	CML212_109_CP	1	0	1	1	0	0	0	0	1	Togasaki et al. Blood Cancer J 2017
	CML212_114_CP	1	0	1	1	0	0	0	0	1	Togasaki et al. Blood Cancer J 2017
	CML212_125_CP	1	0	1	1	0	0	0	0	1	Togasaki et al. Blood Cancer J 2017
	CML212_128_CP	1	0	1	1	0	0	0	0	1	Togasaki et al. Blood Cancer J 2017
	CML212_129_CP	1	0	1	1	0	0	0	0	1	Togasaki et al. Blood Cancer J 2017
	CML212_130_CP	1	0	1	1	0	0	0	0	1	Togasaki et al. Blood Cancer J 2017
	CML212_131_CP	1	0	1	1	0	0	0	0	1	Togasaki et al. Blood Cancer J 2017
	CML212_137_CP	1	0	1	1	0	0	0	0	1	Togasaki et al. Blood Cancer J 2017
	CML212_141_CP	1	0	1	1	0	0	0	0	1	Togasaki et al. Blood Cancer J 2017
	CML212_147_CP	1	0	1	1	0	0	0	0	1	Togasaki et al. Blood Cancer J 2017
	CML212_148_CP	1	0	1	1	0	0	0	0	1	Togasaki et al. Blood Cancer J 2017
	CML212_152_CP	1	0	1	1	0	0	0	0	1	Togasaki et al. Blood Cancer J 2017
	CML212_154_CP	1	0	1	1	0	0	0	0	1	Togasaki et al. Blood Cancer J 2017
	CML212_155_CP	1	0	1	1	0	0	0	0	1	Togasaki et al. Blood Cancer J 2017
	CML212_165_CP	1	0	1	1	0	0	0	0	1	Togasaki et al. Blood Cancer J 2017
	CML212_182_CP	1	0	1	1	0	0	0	0	1	Togasaki et al. Blood Cancer J 2017
	CML212_197_CP	1	0	1	1	0	0	0	0	1	Togasaki et al. Blood Cancer J 2017
	CML212_203_CP	1	0	1	1	0	0	0	0	1	Togasaki et al. Blood Cancer J 2017
	CML212_204_CP	1	0	1	1	0	0	0	0	1	Togasaki et al. Blood Cancer J 2017
	CML212_208_CP	1	0	1	1	0	0	0	0	1	Togasaki et al. Blood Cancer J 2017
	CML212_222_CP	1	0	1	1	0	0	0	0	1	Togasaki et al. Blood Cancer J 2017
	CML212_223_CP	1	0	1	1	0	0	0	0	1	Togasaki et al. Blood Cancer J 2017
	DU01_CP	1	0	1	1	0	0	0	0	1	Mitani et al. Blood 2016
	DU02_CP	1	0	1	1	0	0	0	0	1	Mitani et al. Blood 2016
	DU03_CP	1	0	1	1	0	0	0	0	1	Mitani et al. Blood 2016
	DU04_CP	1	0	1	1	0	0	0	0	1	Mitani et al. Blood 2016
	DU05_CP	1	0	1	1	0	0	0	0	1	Mitani et al. Blood 2016
	DU06_CP	1	0	1	1	0	0	0	0	1	Mitani et al. Blood 2016
	DU07_CP	1	0	1	1	0	0	0	0	1	Mitani et al. Blood 2016
	DU08_CP	1	0	1	1	0	0	0	0	1	Mitani et al. Blood 2016
	DU09_CP	1	0	1	1	0	0	0	0	1	Mitani et al. Blood 2016
	DU10_CP	1	0	1	1	0	0	0	0	1	Mitani et al. Blood 2016
	DU11_CP	1	0	1	1	0	0	0	0	1	Mitani et al. Blood 2016
	DU12_CP	1	0	1	1	0	0	0	0	1	Mitani et al. Blood 2016
	DU13_CP	1	0	1	1	0	0	0	0	1	Mitani et al. Blood 2016
	DU14_CP	1	0	1	1	0	0	0	0	1	Mitani et al. Blood 2016
	DU15_CP	1	0	1	1	0	0	0	0	1	Mitani et al. Blood 2016
	DU16_CP	1	0	1	1	0	0	0	0	1	Mitani et al. Blood 2016
	DU17_CP	1	0	1	1	0	0	0	0	1	Mitani et al. Blood 2016
	DU18_CP	1	0	1	1	0	0	0	0	1	Mitani et al. Blood 2016
	DU19_CP	1	0	1	1	0	0	0	0	1	Mitani et al. Blood 2016
	DU20_CP	1	0	1	1	0	0	0	0	1	Mitani et al. Blood 2016
Case-10-blood		1	1	0	1	0	0	0	0	1	Kim et al. Leuk Res 2017
Case-11-blood		1	1	0	1	0	0	0	0	1	Kim et al. Leuk Res 2017
Case-15-blood		1	1	0	1	0	0	0	0	1	Kim et al. Leuk Res 2017
Case-6-blood		1	1	0	1	0	0	0	0	1	Kim et al. Leuk Res 2017
		216	76	129	85	60	19	112	71	86	

WES: whole-exome sequencing
target: targeted-capture sequencing

Supplementary Table 2: Gene baits for targeted capture sequencing

ABL1	*CEBPA*	*ETV6*	*KIT*	*PDGFRA*	*SETD2*	*UBE2A*
ARID1A	*CHEK2*	*EZH2*	*KLC2*	*PDS5B*	*SF1*	*USP9X*
ARID2	*CREBBP*	*FLT3*	*KMT2A*	*PHF6*	*SF3A1*	*WT1*
ASXL1	*CSF3R*	*GATA1*	*KMT2C*	*PHIP*	*SF3B1*	*ZRSR2*
ASXL2	*CSNK1A1*	*GATA2*	*KMT2D*	*PIGA*	*SMC1A*	
ATRX	*CTCF*	*GNAS*	*KMT2E*	*PPM1D*	*SMC3*	
BCOR	*CUX1*	*GNB1*	*KRAS*	*PRPF8*	*SPI1*	
BCORL1	*DCLRE1C*	*GNB2*	*LUC7L2*	*PTEN*	*SRSF2*	
BRAF	*DDX41*	*HRAS*	*MECOM*	*PTPN1*	*STAG1*	
BRCC3	*DNMT3A*	*IDH1*	*MPL*	*PTPN11*	*STAG2*	
CACNA1H	*DOT1L*	*IDH2*	*MYH11*	*RAD21*	*STAT3*	
CALR	*EED*	*IKZF1*	*NBEAL2*	*RAF1*	*SUZ12*	
CBFB	*EP300*	*IRF1*	*NF1*	*RUNX1*	*TERT*	
CBL	*EPOR*	*JAK1*	*NIPBL*	*SAMD9*	*TET2*	
CBLB	*ETNK1*	*JAK2*	*NOTCH1*	*SAMD9L*	*TP53*	
CCND3	*ETS1*	*KAT6A*	*NPM1*	*SETBP1*	*U2AF1*	
CDKN2A	*ETS2*	*KDM6A*	*NRAS*	*SETD1B*	*U2AF2*	

Supplementary Table 3: Characteristics of patients with CML-CP

Number of cases	148
Age at CP diagnosis (y), median (range) (n = 132)	49 (14-88)
Sex, n (%) (n = 71)	
Male	43 (60.6)
Female	28 (39.4)
TKI use, n (%) (n = 148)	
Yes	115 (77.7)
No	33 (22.3)
Initial TKI, n (%) (n = 60)	
Imatinib	48 (80.0)
Nilotinib	7 (11.7)
Dasatinib	5 (8.3)
Best response to TKI, n (%) (n = 106)	
MMR/CMR	68 (64.2)
CCyR	8 (7.5)
MCyR	10 (9.4)
CHR	19 (17.9)
No haematologic remission	1 (0.9)
Progression to BC, n (%) (n = 148)	
Yes	71 (48.0)
No	77 (52.0)
Time to BC diagnosis (d), median (range) (n = 70)	810 (21-4,653)
Final status, n (%) (n = 71)	
Alive	23 (32.4)
Dead	48 (67.6)
Genotyping method, n (%) (n = 148)	
Whole-exome sequencing	129 (87.2)
Targeted capture sequencing	19 (12.8)
Median follow-up time (m), median (range) (n = 87)	114 (15.3-232)

Supplementary Table 4: Representative CNAs in CML-BC (in-house)

Sample ID	Region	Type of CNA
CML_BC1	*BCR_ABL1*	amplification
CML_BC16	chr8	amplification
CML_BC16	*BCR_ABL1*	amplification
CML_BC16	hyperploidy	amplification
CML_BC17	chr7/7p	deletion
CML_BC17	chr9/9p	deletion
CML_BC17	chr17p	deletion
CML_BC17	hypoploidy	deletion
CML_BC18	*BCR_ABL1*	amplification
CML_BC20	*BCR_ABL1*	amplification
CML_BC20	complex_CNAs	complex_CNAs
CML_BC21	chr19	amplification
CML_BC21	*BCR_ABL1*	amplification
CML_BC22	chr8	amplification
CML_BC22	chr17p	deletion
CML_BC22	*BCR_ABL1*	amplification
CML_BC22	complex_CNAs	complex_CNAs
CML_BC24	chr8	amplification
CML_BC24	chr17p	deletion
CML_BC24	chr19	amplification
CML_BC24	chr21	amplification
CML_BC24	*BCR_ABL1*	amplification
CML_BC24	complex_CNAs	complex_CNAs
CML_BC25	*BCR_ABL1*	amplification
CML_BC3	chr9/9p	deletion
CML_BC3	chr17p	deletion
CML_BC3	*BCR_ABL1*	amplification
CML_BC3	complex_CNAs	complex_CNAs
CML_BC3	*CDKN2A/B*	deletion
CML_BC4	chr7/7p	deletion
CML_BC4	*BCR_ABL1*	amplification
CML_BC8	*BCR_ABL1*	amplification
CML_BC9	chr21	amplification
DU_CML_BC002	chr6	amplification
DU_CML_BC002	chr8	amplification
DU_CML_BC002	chr19	amplification
DU_CML_BC002	chr21	amplification
DU_CML_BC002	*BCR_ABL1*	amplification
DU_CML_BC002	hyperploidy	amplification
DU_CML_BC004	chr17p	deletion
DU_CML_BC004	chr19	amplification
DU_CML_BC004	chr21	amplification
DU_CML_BC004	*BCR_ABL1*	amplification
DU_CML_BC004	complex_CNAs	complex_CNAs
DU_CML_BC005	*RUNX1*	deletion
DU_CML_BC008	chr21	amplification
DU_CML_BC009	chr8	amplification
DU_CML_BC029	*RUNX1*	deletion
GIFU-143-1	chr7/7p	deletion
GIFU-161-1	*RUNX1*	deletion
GIFU-313-1	*IKZF1*	deletion
HCM-017-1	chr8	amplification
KCGH_256_1	chr7/7p	deletion
KCGH203-BC	chr9/9p	deletion

KCGH203-BC	complex_CNAs	complex_CNAs
KCGH-210-1	*IKZF1*	deletion
KCGH-217-1	chr7/7p	deletion
KCGH-350-1	*IKZF1*	deletion
KCGH-762-1	chr17q	amplification
KCGH-762-1	*RUNX1*	deletion
KCGH-762-1	complex_CNAs	complex_CNAs
KCH-116-1	*IKZF1*	deletion
TMU-006-7	*IKZF1*	deletion
TMU-196-1	chr6	amplification
TMU-196-1	*BCR_ABL1*	amplification
TMU-196-1	*IKZF1*	deletion
TMU-298-1	chr8	amplification
TMU-298-1	chr17p	deletion
TMU-960-1	*BCR_ABL1*	amplification
TW-CML-L-002-BC	chr7/7p	deletion
TW-CML-L-003-BC	chr7/7p	deletion
TW-CML-L-003-BC	*RUNX1*	deletion
TW-CML-L-005-BC	*IKZF1*	deletion
TW-CML-L-007-BC	chr7/7p	deletion
TW-CML-L-007-BC	chr9/9p	deletion
TW-CML-L-007-BC	*BCR_ABL1*	amplification
TW-CML-L-007-BC	complex_CNAs	complex_CNAs
TW-CML-L-007-BC	*CDKN2A/B*	deletion
TW-CML-L-007-BC	*IKZF1*	deletion
TW-CML-L-010-BC	chr8	amplification
TW-CML-L-010-BC	chr9/9p	deletion
TW-CML-L-010-BC	complex_CNAs	complex_CNAs
TW-CML-L-010-BC	*CDKN2A/B*	deletion
TW-CML-L-011-BC	chr7/7p	deletion
TW-CML-L-011-BC	chr9/9p	deletion
TW-CML-L-011-BC	chr17p	deletion
TW-CML-L-011-BC	*BCR_ABL1*	amplification
TW-CML-L-011-BC	complex_CNAs	complex_CNAs
TW-CML-L-011-BC	*CDKN2A/B*	deletion
TW-CML-L-012-BC	chr7/7p	deletion
TW-CML-L-012-BC	chr9/9p	deletion
TW-CML-L-012-BC	chr17p	deletion
TW-CML-L-012-BC	*BCR_ABL1*	amplification
TW-CML-L-012-BC	*IKZF1*	deletion
TW-CML-L-012-BC	hypoploidy	deletion
TW-CML-L-013-BC	chr7/7p	deletion
TW-CML-L-013-BC	chr9/9p	deletion
TW-CML-L-013-BC	complex_CNAs	complex_CNAs
TW-CML-L-013-BC	*CDKN2A/B*	deletion
TW-CML-L-014-BC	chr7/7p	deletion
TW-CML-L-014-BC	chr9/9p	deletion
TW-CML-L-014-BC	*BCR_ABL1*	amplification
TW-CML-L-014-BC	complex_CNAs	complex_CNAs
TW-CML-L-014-BC	*CDKN2A/B*	deletion
TW-CML-L-015-BC	chr17p	deletion
TW-CML-L-015-BC	chr17q	amplification
TW-CML-L-015-BC	complex_CNAs	complex_CNAs
TW-CML-L-015-BC	*IKZF1*	deletion
TW-CML-M-001-BC	chr8	amplification
TW-CML-M-001-BC	*BCR_ABL1*	amplification
TW-CML-M-001-BC	complex_CNAs	complex_CNAs

TW-CML-M-006-BC	chr8	amplification
TW-CML-M-006-BC	*BCR_ABL1*	amplification
TW-CML-M-006-BC	complex_CNAs	complex_CNAs
TW-CML-M-007-BC	chr21	amplification
TW-CML-M-007-BC	*RUNX1*	deletion
TW-CML-M-018-BC	*BCR_ABL1*	amplification
TW-CML-M-018-BC	complex_CNAs	complex_CNAs
TW-CML-M-049-BC	chr17q	amplification
TW-CML-M-049-BC	complex_CNAs	complex_CNAs
TW-CML-M-051-BC	chr21	amplification
TW-CML-M-051-BC	complex_CNAs	complex_CNAs
TW-CML-M-051-BC	*RUNX1*	deletion
TW-CML-M-053-BC	chr17p	deletion
TW-CML-M-053-BC	chr19	amplification
TW-CML-M-053-BC	*BCR_ABL1*	amplification
TW-CML-M-053-BC	complex_CNAs	complex_CNAs
TW-CML-M-058-BC	chr19	amplification
TW-CML-M-058-BC	*BCR_ABL1*	amplification
TW-CML-M-058-BC	complex_CNAs	complex_CNAs
TW-CML-M-059-BC	chr19	amplification
TW-CML-M-060-BC	chr6	amplification
TW-CML-M-060-BC	chr8	amplification
TW-CML-M-062-BC	chr6	amplification
TW-CML-M-062-BC	chr8	amplification
TW-CML-M-062-BC	chr17p	deletion
TW-CML-M-062-BC	chr19	amplification
TW-CML-M-062-BC	chr21	amplification
TW-CML-M-062-BC	*BCR_ABL1*	amplification
TW-CML-M-062-BC	hyperploidy	amplification
TW-CML-M-063-BC	chr8	amplification
TW-CML-M-063-BC	chr19	amplification
TW-CML-M-063-BC	chr21	amplification
TW-CML-M-063-BC	hyperploidy	amplification
TW-CML-M-065-BC	chr7/7p	deletion
TW-CML-M-065-BC	complex_CNAs	complex_CNAs
TW-CML-M-066-BC	chr19	amplification
TW-CML-M-066-BC	*BCR_ABL1*	amplification
TW-CML-M-066-BC	complex_CNAs	complex_CNAs
TW-CML-M-069-BC	chr6	amplification
TW-CML-M-069-BC	chr8	amplification
TW-CML-M-069-BC	chr17p	deletion
TW-CML-M-069-BC	chr19	amplification
TW-CML-M-069-BC	chr21	amplification
TW-CML-M-069-BC	*BCR_ABL1*	amplification
TW-CML-M-069-BC	hyperploidy	amplification
TW-CML-M-070-BC	chr8	amplification
TW-CML-M-070-BC	chr17q	amplification
TW-CML-M-070-BC	complex_CNAs	complex_CNAs
TW-CML-M-074-BC	chr17p	deletion
TW-CML-M-074-BC	chr17q	amplification
TW-CML-M-074-BC	complex_CNAs	complex_CNAs
TW-CML-M-075-BC	*RUNX1*	deletion
TW-CML-M-076-BC	chr17p	deletion
TW-CML-M-076-BC	complex_CNAs	complex_CNAs
TW-CML-M-078-BC	chr6	amplification
TW-CML-M-078-BC	chr8	amplification
TW-CML-M-078-BC	chr19	amplification

TW-CML-M-078-BC	chr21	amplification
TW-CML-M-078-BC	hyperploidy	amplification
TW-CML-M-079-BC	chr8	amplification
TW-CML-M-080-BC	*RUNX1*	deletion
TW-CML-M-081-BC	chr8	amplification
TW-CML-M-081-BC	complex_CNAs	complex_CNAs
TW-CML-M-081-BC	*RUNX1*	deletion
TW-CML-M-082-BC	chr8	amplification
TW-CML-M-082-BC	chr21	amplification
TW-CML-M-082-BC	*BCR_ABL1*	amplification
TW-CML-M-082-BC	complex_CNAs	complex_CNAs
TW-CML-M-083-BC	*BCR_ABL1*	amplification
TW-CML-M-083-BC	complex_CNAs	complex_CNAs
TW-CML-M-086-BC	chr7/7p	deletion
TW-CML-M-086-BC	chr8	amplification
TW-CML-M-086-BC	chr9/9p	deletion
TW-CML-M-086-BC	complex_CNAs	complex_CNAs
TW-CML-M-086-BC	*CDKN2A/B*	deletion
AKT-001	*BCR_ABL1*	amplification
AKT-003	chr8	amplification
AKT-003	chr17p	deletion
AKT-003	chr17q	amplification
AKT-005	chr17p	deletion
AKT-005	chr17q	amplification
AKT-006	chr7/7p	deletion
AKT-006	chr19	amplification
AKT-006	*BCR_ABL1*	amplification
AKT-006	complex_CNAs	complex_CNAs
JUN-001	*BCR_ABL1*	amplification
JUN-001	*IKZF1*	deletion
JUN-002	chr9/9p	deletion
JUN-002	*IKZF1*	deletion
JUN-002	*CDKN2A/B*	deletion
JUN-002	*RUNX1*	deletion
JUN-002	complex_CNAs	complex_CNAs
JUN-003	hypoploidy	deletion
JUN-003	chr7/7p	deletion
JUN-003	chr9/9p	deletion
JUN-003	*BCR_ABL1*	amplification
JUN-003	*CDKN2A/B*	deletion
JUN-003	complex_CNAs	complex_CNAs

Supplementary Table 5: Characteristics of patients with CML-BC analysed for survival

	Internal cohort	External cohort	*P*
Number of cases (n =116)	99	17	
Age at BC diagnosis (y), median (range) (n = 116)	50 (16-86)	55 (19-84)	0.60
Sex, n (%) (n = 116)			1
Male	59 (59.6)	10 (58.8)	
Female	40 (40.4)	7 (41.2)	
Lineage of blasts, n (%) (n = 116)			0.79
Myeloid	64 (64.6)	12 (70.6)	
Lymphoid	35 (35.4)	5 (29.4)	
WBC ($\times 10^3$/uL), median (range) (n = 116)	50,100 (1,700-412,000)	11,000 (2,220-580,000)	0.094
Hb (g/dL), median (range) (n = 116)	9.3 (5.0-15.8)	11.8 (7.7-15.3)	0.014
PLT ($\times 10^3$/uL), median (range) (n = 116)	96,000 (3,000-2,740,000)	133,000 (10,000-496,000)	0.24
LDH (U/L), median (range) (n = 75)	850 (75-6,332)	465 (189-3,671)	0.032
Blast in BM (%), median (range) (n = 113)	59 (1.0-98)	50 (17-96)	0.41
Prior history of CP diagnosis, n (%) (n = 115)			1.00
Yes	76 (77.6)	14 (82.4)	
No	22 (22.4)	3 (17.6)	
Time from CP diagnosis (m), median (range) (n = 85)	34.3 (0.27-363)	35.2 (4.0-98.4)	0.64
Age at CP diagnosis (y), median (range) (n = 85)	43 (14-85)	48 (14-82)	0.26
Prior TKI therapy before BC, n (%) (n = 114)			0.11
Yes	36 (37.1)	10 (58.8)	
No	61 (62.9)	7 (41.2)	
TKIs used for CP, n (%) (n = 46)			1
Imatinib	34 (94.4)	10 (100)	
Dasatinib	2 (5.6)	0 (0)	
TKI-based therapy for BC, n (%) (n = 116)			0.43
Yes	59 (59.6)	12 (70.6)	
No	40 (40.4)	5 (29.4)	
TKIs used for BC, n (%) (n = 71)			0.11
Imatinib	33 (55.9)	3 (25.0)	
Dasatinib	23 (39.0)	8 (66.7)	
Nilotinib	2 (3.4)	0	
Ponatinib	1 (1.7)	1 (8.3)	
Final status, n (%) (n = 116)			0.12
Alive	24 (24.2)	1 (5.9)	
Dead	75 (75.8)	16 (94.1)	
Method, n (%) (n = 116)			<0.001
Whole-exome sequencing	50 (50.5)	17 (100)	
Targeted capture sequencing	49 (49.5)	0 (0)	
Median follow-up time (y), median (range) (n = 116)	3.2 (0.48-30.4)	12.9 (12.9-12.9)	0.33

The two-sided Wilcoxon rank-sum test and Fisher's exact test were used for continuous and categorical data to calculate *P*-values, respectively.

Supplementary Table 6: Representative CNAs in CML-CP (in-house)

Sample ID	Region	Type of CNA
TW_CML_M_001_D	chr8	amplification
TW_CML_M_078_D	*BCR_ABL1*	amplification
TW_CML_M_080_D	*RUNX1*	deletion

Supplementary Table 7: Primers used in this study

Name	Sequence	Application
RUNX1-ETS2-QF	CTTCACAAACCCACCGCAAG	measurement of *RUNX1-ETS2*
RUNX1-ETS2-QR	AGGGAGTCTGAGCTCTCGAAG	measurement of *RUNX1-ETS2*
1_CML_chr1_11189760+300_F	AAGCGGCCGCGCCTACCAGAGTTGCATCCT	deep amplicon-sequencing
2_CML_chr1_38187211+287_F	AAGCGGCCGCAGCTCCCTCCCATAGCTGA	deep amplicon-sequencing
3_CML_chr1_115258621+279_F	AAGCGGCCGCCCGACAAGTGAGAGACAGGA	deep amplicon-sequencing
4_CML_chr2_209112973+260_F	AAGCGGCCGCTCATACCTTGCTTAATGGGTGT	deep amplicon-sequencing
5_CML_chr3_10089560+290_F	AAGCGGCCGCTCCTACAGCTTCTTTTCTCTCTCT	deep amplicon-sequencing
6_CML_chr3_12632297+250_F	AAGCGGCCGCTCCATTCCCTGAGCCGTCT	deep amplicon-sequencing
7_CML_chr3_47036851+278_F	AAGCGGCCGCATTGGACTCGGAACCAGGC	deep amplicon-sequencing
8_CML_chr3_47043874+247_F	AAGCGGCCGCTGTCCCAGTTCGAAATGGACA	deep amplicon-sequencing
9_CML_chr3_47165521+300_F	AAGCGGCCGCAGTGTTGTGGCTTGGGCA	deep amplicon-sequencing
10_CML_chr3_128200592+296_F	AAGCGGCCGCTCTTGCCTGGCAGCACAA	deep amplicon-sequencing
11_CML_chr3_128204756+275_F	AAGCGGCCGCGGGGACTGCCACTTTCCAT	deep amplicon-sequencing
12_CML_chr4_106156656+250_F	AAGCGGCCGCTGGTAGCAGTGGAGAGCT	deep amplicon-sequencing
13_CML_chr4_106157416+250_F	AAGCGGCCGCGGATCATTCTTTGGCCAGACT	deep amplicon-sequencing
14_CML_chr5_176939030+279_F	AAGCGGCCGCTCTGACTTCCAGCACCCCT	deep amplicon-sequencing
15_CML_chr6_56358814+250_F	AAGCGGCCGCTTCATTGAGTTTGGTTTCCACC	deep amplicon-sequencing
16_CML_chr6_79694871+465_F	AAGCGGCCGCGCAGATCTAGTTGCAATCACCA	deep amplicon-sequencing
17_CML_chr6_79728662+333_F	AAGCGGCCGCTCTCAGTTACTTATTAGCTCTGGCA	deep amplicon-sequencing
18_CML_chr6_117680739+376_F	AAGCGGCCGCCCCAGGCTCTAACACAGCA	deep amplicon-sequencing
19_CML_chr7_50467801+349_F	AAGCGGCCGCCCGATGTACCAGCTGCACA	deep amplicon-sequencing
20_CML_chr7_50468003+245_F	AAGCGGCCGCACCTGACCAACCACATCGC	deep amplicon-sequencing
21_CML_chr7_101918419+385_F	AAGCGGCCGCTTGGCAGAAATCCCCCAGG	deep amplicon-sequencing
22_CML_chr7_104681328+320_F	AAGCGGCCGCTGGCATGCCCCAGTGTTT	deep amplicon-sequencing
23_CML_chr7_151945032+478_F	AAGCGGCCGCAGCTGGTTTACCCATGCCA	deep amplicon-sequencing
24_CML_chr8_128753053+250_F	AAGCGGCCGCCCCCCAAGGTAGTTATCCTTAAA	deep amplicon-sequencing
25_CML_chr9_133738182+299_F	AAGCGGCCGCCCTGGCCGAGTTGGTTCAT	deep amplicon-sequencing
26_CML_chr9_133747460+248_F	AAGCGGCCGCACCCACTGAAAAGCACTTCCT	deep amplicon-sequencing
27_CML_chr9_133748121+283_F	AAGCGGCCGCTGTTGGAAGTTGGGCCCA	deep amplicon-sequencing
28_CML_chr9_133760457+250_F	AAGCGGCCGCTCCTGGGCGCAAAGACAA	deep amplicon-sequencing
29_CML_chr10_45485034+291_F	AAGCGGCCGCGGGTGTAGTTACTGTCAGGGG	deep amplicon-sequencing
30_CML_chr10_112343854+271_F	AAGCGGCCGCTGTTTCTGTGTGCAGTTGACA	deep amplicon-sequencing
31_CML_chr10_124271411+247_F	AAGCGGCCGCGGTGAGAGCTGAGTTTTGCG	deep amplicon-sequencing
32_CML_chr11_32414106+274_F	AAGCGGCCGCACACATGGCTGACTCTCTCA	deep amplicon-sequencing
33_CML_chr11_32417788+249_F	AAGCGGCCGCAGCGGGCACACTTACCAGT	deep amplicon-sequencing
34_CML_chr11_66031296+242_F	AAGCGGCCGCAGAAGCTTCCGTTCTCCCA	deep amplicon-sequencing
35_CML_chr11_66033292+291_F	AAGCGGCCGCATTCACTGCCTGATGCCCC	deep amplicon-sequencing
36_CML_chr11_66033805+244_F	AAGCGGCCGCACCCACCAGTCTGTTCCCT	deep amplicon-sequencing
37_CML_chr12_4409025+276_F	AAGCGGCCGCGGATTGTCTCAAAGCTTGCCA	deep amplicon-sequencing
38_CML_chr12_49441667+296_F	AAGCGGCCGCTGGTATGGCCAGGACAAGG	deep amplicon-sequencing
39_CML_chr12_69233300+248_F	AAGCGGCCGCACACAAGCTTCACAATCACAAG	deep amplicon-sequencing
40_CML_chr12_112888090+250_F	AAGCGGCCGCCCCTTGCCTCCCTTTCCAA	deep amplicon-sequencing
41_CML_chr12_112926085+297_F	AAGCGGCCGCGCCCTATGCTTTTTGCCAACA	deep amplicon-sequencing
42_CML_chr12_112926734+279_F	AAGCGGCCGCAGCATTGTCTCTGAGTCCACT	deep amplicon-sequencing
43_CML_chr12_122243732+240_F	AAGCGGCCGCCCCTGCCAGATCGATGAGT	deep amplicon-sequencing
44_CML_chr12_122255571+398_F	AAGCGGCCGCCCAGCTCTCCTCCTCCTCAA	deep amplicon-sequencing
45_CML_chr12_122263104+276_F	AAGCGGCCGCACCCACCAGCCTCTCTTCA	deep amplicon-sequencing
46_CML_chr14_45628321+134_F	AAGCGGCCGCACGTGATGAGACCCGAGTT	deep amplicon-sequencing
47_CML_chr15_90631786+280_F	AAGCGGCCGCAGGTCAGTGGATCCCCTCTC	deep amplicon-sequencing
48_CML_chr15_90631786+280_F	AAGCGGCCGCAGGTCAGTGGATCCCCTCTC	deep amplicon-sequencing
49_CML_chr16_3781226+296_F	AAGCGGCCGCGTGGTGCTTGCACTCGTTG	deep amplicon-sequencing
50_CML_chr16_3817599+296_F	AAGCGGCCGCGGCAAGAAAGGTAAAAGGGCA	deep amplicon-sequencing
51_CML_chr16_15818442+293_F	AAGCGGCCGCGGGATCTCAGCGCAGAGAA	deep amplicon-sequencing
52_CML_chr16_24573255+244_F	AAGCGGCCGCACAACCTCTGATGAGATCTCCG	deep amplicon-sequencing
53_CML_chr16_67670562+265_F	AAGCGGCCGCCACCACCTGTGCTTCCTGA	deep amplicon-sequencing
54_CML_chr17_7577411+272_F	AAGCGGCCGCAATCGGTAAGAGGTGGGCC	deep amplicon-sequencing
55_CML_chr17_7578082+297_F	AAGCGGCCGCAAGCAGCAGGAGAAAGCCC	deep amplicon-sequencing
56_CML_chr17_37566570+296_F	AAGCGGCCGCATCGAAGAGACAGGTGGCG	deep amplicon-sequencing
57_CML_chr18_29099587+382_F	AAGCGGCCGCAGGCCCTATGCAGTTTGCT	deep amplicon-sequencing
58_CML_chr20_31021575+241_F	AAGCGGCCGCAATCCTTTGAGCAGGCGG	deep amplicon-sequencing
59_CML_chr20_31022179+482_F	AAGCGGCCGCTCCCTAGGTCAGATCACCCA	deep amplicon-sequencing
60_CML_chr20_31022674+250_F	AAGCGGCCGCACCTGCCTTCTCTGAGAAAGG	deep amplicon-sequencing
61_CML_chr20_49195660+243_F	AAGCGGCCGCTGAGAATTGGACCTGGCTGAC	deep amplicon-sequencing
62_CML_chr20_57415469+290_F	AAGCGGCCGCACGAGGAAGAGTTCGACTACG	deep amplicon-sequencing
63_CML_chr21_30927357+300_F	AAGCGGCCGCGAAAGAGATGACTCACCTGTTCA	deep amplicon-sequencing
64_CML_chr21_36164269+400_F	AAGCGGCCGCGCCTGACCTACAGCGAGATC	deep amplicon-sequencing
65_CML_chr21_36231667+250_F	AAGCGGCCGCGGGAAAGGTTGAACCCAAGGA	deep amplicon-sequencing

66_CML_chr21_36252741+244_F	AAGCGGCCGCTGTTAAGACAGACCGAGTTTCT	deep amplicon-sequencing
67_CML_chr21_36252801+283_F	AAGCGGCCGCTGGGTTTGTTGCCATGAAACG	deep amplicon-sequencing
68_CML_chr21_36258989+363_F	AAGCGGCCGCTCCCCCACATCCCAAGCTA	deep amplicon-sequencing
69_CML_chrX_39922923+250_F	AAGCGGCCGCGCCCTTTTCCTGCCAGGTT	deep amplicon-sequencing
70_CML_chrX_39923662+248_F	AAGCGGCCGCGCTGCTGTCACCTGAGACT	deep amplicon-sequencing
71_CML_chrX_39931966+276_F	AAGCGGCCGCACGGTGAAGACTGGCTGT	deep amplicon-sequencing
72_CML_chrX_39933746+289_F	AAGCGGCCGCTGTCTGCGCAATGGACGA	deep amplicon-sequencing
73_CML_chrX_41025250+273_F	AAGCGGCCGCTGATGGGGGATGAACCAGAC	deep amplicon-sequencing
74_CML_chrX_44942614+316_F	AAGCGGCCGCTCTGATTGGAACACAAGGGTT	deep amplicon-sequencing
75_CML_chrX_53441831+272_F	AAGCGGCCGCTGCATGAGTTGGCAAGGGT	deep amplicon-sequencing
76_CML_chrX_118708577+295_F	AAGCGGCCGCCCTCCCCTTCTCCTGCTTCT	deep amplicon-sequencing
77_CML_chrX_118708697+370_F	AAGCGGCCGCGCCTCATGCGGGACTTCAA	deep amplicon-sequencing
78_CML_chrX_118716950+298_F	AAGCGGCCGCAGCAGATTCACATAACTCTGGGT	deep amplicon-sequencing
79_CML_chrX_129148413+300_F	AAGCGGCCGCTGCTTCTGCCAAGGTGCT	deep amplicon-sequencing
80_CML_chrX_129149659+240_F	AAGCGGCCGCGCTTGTGGCCTGAAGCT	deep amplicon-sequencing
81_CML_chrX_133548839+566_F	AAGCGGCCGCGGCAGTCCTCATTTTATTTCAATGTCC	deep amplicon-sequencing
1_CML_chr1_11189760+300_R	AAGCGGCCGCGTCTTTGCTATGTGCCAGGT	deep amplicon-sequencing
2_CML_chr1_38187211+287_R	AAGCGGCCGCTGTGGCTGCTTGCAGCT	deep amplicon-sequencing
3_CML_chr1_115258621+279_R	AAGCGGCCGCTGGAAGGTCACACTAGGGT	deep amplicon-sequencing
4_CML_chr2_209112973+260_R	AAGCGGCCGCGGAAATCACCAAATGGCACCA	deep amplicon-sequencing
5_CML_chr3_10089560+290_R	AAGCGGCCGCAGACCCAGGTCAGAGTTCCT	deep amplicon-sequencing
6_CML_chr3_12632297+250_R	AAGCGGCCGCTCCACGGGAAAGCACAGT	deep amplicon-sequencing
7_CML_chr3_47036851+278_R	AAGCGGCCGCCCCACCTGTACAGCTGCTT	deep amplicon-sequencing
8_CML_chr3_47043874+247_R	AAGCGGCCGCATAGGTCAGTCCCCTGGCT	deep amplicon-sequencing
9_CML_chr3_47165521+300_R	AAGCGGCCGCCCCTCCAGCTGTACCTCTTC	deep amplicon-sequencing
10_CML_chr3_128200592+296_R	AAGCGGCCGCTTGACTGAGCTGGTGGGGA	deep amplicon-sequencing
11_CML_chr3_128204756+275_R	AAGCGGCCGCACTCTCTGTGTACCCAGGGG	deep amplicon-sequencing
12_CML_chr4_106156656+250_R	AAGCGGCCGCGGAGGTCATTTGATTGGAGAGA	deep amplicon-sequencing
13_CML_chr4_106157416+250_R	AAGCGGCCGCCTGCAAAAAGTTCAGGATGTGT	deep amplicon-sequencing
14_CML_chr5_176939030+279_R	AAGCGGCCGCAGCGCCACCTTAGCTGTT	deep amplicon-sequencing
15_CML_chr6_56358814+250_R	AAGCGGCCGCGAGCTTGGGACTGATGACCT	deep amplicon-sequencing
16_CML_chr6_79694871+465_R	AAGCGGCCGCTCTGCCCACCTTGACCTCT	deep amplicon-sequencing
17_CML_chr6_79728662+333_R	AAGCGGCCGCAGGGAGCCATCTGTTGCT	deep amplicon-sequencing
18_CML_chr6_117680739+376_R	AAGCGGCCGCTGGATCAAAATCAAGTTGTGTGGAC	deep amplicon-sequencing
19_CML_chr7_50467801+349_R	AAGCGGCCGCACACCTTCATCTGCTCCCC	deep amplicon-sequencing
20_CML_chr7_50468003+245_R	AAGCGGCCGCCATGTTGCACTCAAAAGGATCAC	deep amplicon-sequencing
21_CML_chr7_101918419+385_R	AAGCGGCCGCTCAAAGCCCAGTGTGGCA	deep amplicon-sequencing
22_CML_chr7_104681328+320_R	AAGCGGCCGCAGCAATCACGTTCTCAGTGTT	deep amplicon-sequencing
23_CML_chr7_151945032+478_R	AAGCGGCCGCAGAGGAACCTGAAACAGTGGT	deep amplicon-sequencing
24_CML_chr8_128753053+250_R	AAGCGGCCGCAAGGTTGTGAGGTTGCATTTG	deep amplicon-sequencing
25_CML_chr9_133738182+299_R	AAGCGGCCGCAATGCCAGCAGACGCCTT	deep amplicon-sequencing
26_CML_chr9_133747460+248_R	AAGCGGCCGCTCCAACGAGGTTTTGTGCAG	deep amplicon-sequencing
27_CML_chr9_133748121+283_R	AAGCGGCCGCTCCAGGTACTCCATGGCTGA	deep amplicon-sequencing
28_CML_chr9_133760457+250_R	AAGCGGCCGCACTCGGGTTGATATGAGAGGGA	deep amplicon-sequencing
29_CML_chr10_45485034+291_R	AAGCGGCCGCTTCATGCTGGGAGGCACAG	deep amplicon-sequencing
30_CML_chr10_112343854+271_R	AAGCGGCCGCTGCTTCAGTGTCTTCCAAATCC	deep amplicon-sequencing
31_CML_chr10_124271411+247_R	AAGCGGCCGCAACAGCGCTCCCTCTTCCT	deep amplicon-sequencing
32_CML_chr11_32414106+274_R	AAGCGGCCGCTCCTAGTAGGAGAGGTTGCCT	deep amplicon-sequencing
33_CML_chr11_32417788+249_R	AAGCGGCCGCTCCAGTGCTCACTCTCCCT	deep amplicon-sequencing
34_CML_chr11_66031296+242_R	AAGCGGCCGCTGGGTGAAACTTGCCCAGG	deep amplicon-sequencing
35_CML_chr11_66033292+291_R	AAGCGGCCGCTCAAGGAGCCACTGCCATC	deep amplicon-sequencing
36_CML_chr11_66033805+244_R	AAGCGGCCGCTGAAGAGGCATGCTGGAGG	deep amplicon-sequencing
37_CML_chr12_4409025+276_R	AAGCGGCCGCTGATCTAGGTGGGGGCAGA	deep amplicon-sequencing
38_CML_chr12_49441667+296_R	AAGCGGCCGCTGGGGTTCCTGACTCTGGT	deep amplicon-sequencing
39_CML_chr12_69233300+248_R	AAGCGGCCGCGCACATGTAAAGCAGGCCA	deep amplicon-sequencing
40_CML_chr12_112888090+250_R	AAGCGGCCGCGTGGTCACTAAAATGTTACTGACCT	deep amplicon-sequencing
41_CML_chr12_112926085+297_R	AAGCGGCCGCTCGTGAGCACTTTCCTTCCA	deep amplicon-sequencing
42_CML_chr12_112926734+279_R	AAGCGGCCGCTGAGAATCCGCATGCCAG	deep amplicon-sequencing
43_CML_chr12_122243732+240_R	AAGCGGCCGCGGAAGTGCTGTGCAAGTGC	deep amplicon-sequencing
44_CML_chr12_122255571+398_R	AAGCGGCCGCAGATGGAACCGTACGCCAC	deep amplicon-sequencing
45_CML_chr12_122263104+276_R	AAGCGGCCGCCTCTGGCCTCACAACTCCTC	deep amplicon-sequencing
46_CML_chr14_45628321+134_R	AAGCGGCCGCTCGTGCTTTTCCCTGAGGC	deep amplicon-sequencing
47_CML_chr15_90631786+280_R	AAGCGGCCGCCGTCTGGCTGTGTTGTTGC	deep amplicon-sequencing
48_CML_chr15_90631786+280_R	AAGCGGCCGCCGTCTGGCTGTGTTGTTGC	deep amplicon-sequencing
49_CML_chr16_3781226+296_R	AAGCGGCCGCAGGTGCCATGTCCCTTGTG	deep amplicon-sequencing
50_CML_chr16_3817599+296_R	AAGCGGCCGCTGCAAGGAGCTTCCCAAGT	deep amplicon-sequencing
51_CML_chr16_15818442+293_R	AAGCGGCCGCTGCCACATCATCCAGGGGA	deep amplicon-sequencing
52_CML_chr16_24573255+244_R	AAGCGGCCGCAAACACACAGGCTTACCGAA	deep amplicon-sequencing
53_CML_chr16_67670562+265_R	AAGCGGCCGCACAAGGACCCATCTGGCTC	deep amplicon-sequencing
54_CML_chr17_7577411+272_R	AAGCGGCCGCAAAAGGCCTCCCCTGCTTG	deep amplicon-sequencing

55_CML_chr17_7578082+297_R	AAGCGGCCGCTAGCGATGGTGAGCAGCTG	deep amplicon-sequencing
56_CML_chr17_37566570+296_R	AAGCGGCCGCAAAAAGAACCTGCCCCCGG	deep amplicon-sequencing
57_CML_chr18_29099587+382_R	AAGCGGCCGCACATAAAAGTCCTCTCACACCACA	deep amplicon-sequencing
58_CML_chr20_31021575+241_R	AAGCGGCCGCTTAACTTCAGGGCCCCAGA	deep amplicon-sequencing
59_CML_chr20_31022179+482_R	AAGCGGCCGCTCTGGACATGGCAGTTCCG	deep amplicon-sequencing
60_CML_chr20_31022674+250_R	AAGCGGCCGCTGCTCCTCATCATCACTTTCCC	deep amplicon-sequencing
61_CML_chr20_49195660+243_R	AAGCGGCCGCTGGTCACACCTGGATTCAAACA	deep amplicon-sequencing
62_CML_chr20_57415469+290_R	AAGCGGCCGCTTTGTCCTCGGGCTTGAGC	deep amplicon-sequencing
63_CML_chr21_30927357+300_R	AAGCGGCCGCTGGTGACCAATATCCAGATGG	deep amplicon-sequencing
64_CML_chr21_36164269+400_R	AAGCGGCCGCTACCACCTGTACTACGGCG	deep amplicon-sequencing
65_CML_chr21_36231667+250_R	AAGCGGCCGCACCAACCTCATTCTGTTTTGTTCTC	deep amplicon-sequencing
66_CML_chr21_36252741+244_R	AAGCGGCCGCTGGCACTCTGGTCACTGT	deep amplicon-sequencing
67_CML_chr21_36252801+283_R	AAGCGGCCGCCACTACACAAATGCCCTAAAAGTGT	deep amplicon-sequencing
68_CML_chr21_36258989+363_R	AAGCGGCCGCTGAGCCCAGGCAAGATGAG	deep amplicon-sequencing
69_CML_chrX_39922923+250_R	AAGCGGCCGCCCTCTAACCACTTAGAAGACCCAC	deep amplicon-sequencing
70_CML_chrX_39923662+248_R	AAGCGGCCGCGCCTTCTCATGGCGACCTT	deep amplicon-sequencing
71_CML_chrX_39931966+276_R	AAGCGGCCGCGCCGAACCCCAACTGGAAT	deep amplicon-sequencing
72_CML_chrX_39933746+289_R	AAGCGGCCGCCTGCCCTGGGTCAATCCTT	deep amplicon-sequencing
73_CML_chrX_41025250+273_R	AAGCGGCCGCCGTACATGAAAAAGTACATCAGACACC	deep amplicon-sequencing
74_CML_chrX_44942614+316_R	AAGCGGCCGCTCTGGCTGTCTTTGCATGT	deep amplicon-sequencing
75_CML_chrX_53441831+272_R	AAGCGGCCGCGGATGCCATCAGCTTTGTGC	deep amplicon-sequencing
76_CML_chrX_118708577+295_R	AAGCGGCCGCCCTGCAACCTTCGGGAAGA	deep amplicon-sequencing
77_CML_chrX_118708697+370_R	AAGCGGCCGCCCCAGCCAGACCCAAACAT	deep amplicon-sequencing
78_CML_chrX_118716950+298_R	AAGCGGCCGCTGGCCAGCTTCTTTAAACTGT	deep amplicon-sequencing
79_CML_chrX_129148413+300_R	AAGCGGCCGCTTGCTGCTGGTGTGCAC	deep amplicon-sequencing
80_CML_chrX_129149659+240_R	AAGCGGCCGCTTTCACTCGCCCCAGATCC	deep amplicon-sequencing
81_CML_chrX_133548839+566_R	AAGCGGCCGCACGGCTTGCAAATGCCTTG	deep amplicon-sequencing

Supplementary Table 8: Public datasets used in this study

Datasets	Accession ID
Kim, T. et al. Leuk Res 59, 142-148 (2017).	PRJEB20846
Branford, S. et al. Blood 132, 948-961 (2018).	EGAS00001003071
