# Supplementary material for: Clonal evolution and clinical implications of genetic abnormalities in blastic transformation of chronic myeloid leukaemia
Source: Nat Commun. 2021 May 14;12:2833. doi: 10.1038/s41467-021-23097-w (PMC8121838; doi:10.1038/s41467-021-23097-w)
Supplement: Supplementary file 2 — Description of Additional Supplementary Files [file 41467_2021_23097_MOESM2_ESM.pdf]

Description of Additional Supplementary Files

File Name: Supplementary Data 1
Description: Summary of mutations in CML-BC (in-house)

File Name: Supplementary Data 2
Description: Summary of mutations in CML-CP (in-house)
