# Supplementary material for: Clonal evolution and clinical implications of genetic abnormalities in blastic transformation of chronic myeloid leukaemia
Source: Nat Commun. 2021 May 14;12:2833. doi: 10.1038/s41467-021-23097-w (PMC8121838; doi:10.1038/s41467-021-23097-w)
Supplement: Supplementary file 4 — Reporting Summary [file 41467_2021_23097_MOESM4_ESM.pdf]

nature research

Corresponding author(s): Lee-Yung Shih, Seishi Ogawa

Last updated by author(s): 2021/04/13

Reporting Summary

Nature Research wishes to improve the reproducibility of the work that we publish. This form provides structure for consistency and transparency in reporting. For further information on Nature Research policies, see our Editorial Policies and the Editorial Policy Checklist.

Statistics

For all statistical analyses, confirm that the following items are present in the figure legend, table legend, main text, or Methods section.

n/a	Confirmed	
☐	☒	The exact sample size (n) for each experimental group/condition, given as a discrete number and unit of measurement
☐	☒	A statement on whether measurements were taken from distinct samples or whether the same sample was measured repeatedly
☐	☒	The statistical test(s) used AND whether they are one- or two-sided<br>*Only common tests should be described solely by name; describe more complex techniques in the Methods section.*
☐	☒	A description of all covariates tested
☐	☒	A description of any assumptions or corrections, such as tests of normality and adjustment for multiple comparisons
☐	☒	A full description of the statistical parameters including central tendency (e.g. means) or other basic estimates (e.g. regression coefficient) AND variation (e.g. standard deviation) or associated estimates of uncertainty (e.g. confidence intervals)
☐	☒	For null hypothesis testing, the test statistic (e.g. F, t, r) with confidence intervals, effect sizes, degrees of freedom and P value noted<br>*Give P values as exact values whenever suitable.*
☒	☐	For Bayesian analysis, information on the choice of priors and Markov chain Monte Carlo settings
☒	☐	For hierarchical and complex designs, identification of the appropriate level for tests and full reporting of outcomes
☒	☐	Estimates of effect sizes (e.g. Cohen's d, Pearson's r), indicating how they were calculated

Our web collection on statistics for biologists contains articles on many of the points above.

Software and code

Policy information about availability of computer code

Data collection	No software was used for data collection.
Data analysis	Detailed descriptions of the software and analysis are provided in the Method section. Briefly, sequence alignment and mutation calling were performed using hg19 reference genome and Genomon pipeline (https://github.com/Genomon-Project). CNAs and SVs were detected using the CNACS algorithm and Genomon-SV pipeline, respectively. Statistical analyses were performed using R (v3.5.0).<br><br>List of programs and softwares:<br>Genomon 2 pipeline: version 2.6.2 (https://genomon.readthedocs.io/ja/latest/)<br>Integrative Genomics Viewer (IGV): version 2.3.97 (https://software.broadinstitute.org/software/igv/)<br>CNACS: (http://plaza.umin.ac.jp/kyoto_tumorpatho/CNACS.html)<br>R: version 3.5.0 (https://www.r-project.org/)<br>ExomeDepth: version 1.1.15 (https://cran.r-project.org/web/packages/ExomeDepth/index.html)<br>rms: version 6.2-0 (https://cran.r-project.org/web/packages/rms/index.html)

For manuscripts utilizing custom algorithms or software that are central to the research but not yet described in published literature, software must be made available to editors and reviewers. We strongly encourage code deposition in a community repository (e.g. GitHub). See the Nature Research guidelines for submitting code & software for further information.

Data

Policy information about availability of data

All manuscripts must include a data availability statement. This statement should provide the following information, where applicable:

- Accession codes, unique identifiers, or web links for publicly available datasets
- A list of figures that have associated raw data
- A description of any restrictions on data availability

The WES data in this study are deposited in the European Genome-phenome Archive under accession code EGAS00001005075. The data is available under restricted access, and access can be obtained by contacting Seishi Ogawa (sogawa-tky@umin.ac.jp). The public WES data used in this study are available in the European Genome-phenome Archive under accession code EGAS00001003071, and the European Nucleotide Archive under accession code PRJEB20846 (Supplementary Table 8). The remaining data are available within the Article, Supplementary Information or available from the authors upon request.

Field-specific reporting

Please select the one below that is the best fit for your research. If you are not sure, read the appropriate sections before making your selection.

☒ Life sciences ☐ Behavioural & social sciences ☐ Ecological, evolutionary & environmental sciences

For a reference copy of the document with all sections, see nature.com/documents/nr-reporting-summary-flat.pdf

Life sciences study design

All studies must disclose on these points even when the disclosure is negative.

Sample size	No statistical methods were used to determine sample size since this is an exploratory study. We performed WES, targeted-capture sequencing, and/or deep amplicon-sequencing using 112 CML-BC and 71 CP samples at diagnosis from 130 patients at ten institutions enrolled in this study. Combined with the external WES data of 24 BC and 77 CP patients, we comprehensively analysed a total of 136 BC and 148 CP samples obtained from 216 CML patients. The sample size of this study was sufficient to confirm novel findings with statistical significance.
Data exclusions	To analyse the number of SNVs acquired during disease progression (Fig. 1b and Supplementary Fig. 1b, d), WES data of both CP and BC samples were available for 15 patients for the external cohort. Of these, 13 were subjected to analysis after excluding 1 case which lacked information on progression time from CP to BC and 1 in which a much lower depth was observed compared to the other samples.
Replication	Putative driver mutations detected by WES were subjected to the validation using deep amplicon-sequencing in both CP and BC samples as described previously, and mutations with VAF ≥ 0.02 were considered to be validated.
Randomization	Not applicable since this is a case-series study, which was not planned to detect any difference in effects between the cohorts with and without intervention.
Blinding	Not applicable since this is a case-series study, which was not planned to detect any difference in effects between the cohorts with and without intervention.

Reporting for specific materials, systems and methods

We require information from authors about some types of materials, experimental systems and methods used in many studies. Here, indicate whether each material, system or method listed is relevant to your study. If you are not sure if a list item applies to your research, read the appropriate section before selecting a response.

Materials & experimental systems

n/a	Involved in the study	
☒	☐	Antibodies
☒	☐	Eukaryotic cell lines
☒	☐	Palaeontology and archaeology
☒	☐	Animals and other organisms
☐	☒	Human research participants
☒	☐	Clinical data
☒	☐	Dual use research of concern

Methods

n/a	Involved in the study	
☒	☐	ChIP-seq
☒	☐	Flow cytometry
☒	☐	MRI-based neuroimaging

Human research participants

Policy information about studies involving human research participants

Population characteristics	We obtained 112 CML-BC and 71 CP samples from 130 patients at ten institutions in Japan or Taiwan enrolled in this study. Detailed information are available in Table 1 and Supplementary Table 3.
Recruitment	To analyse clonal evolution of CML, 52 patients with available CP and BC samples were selectively enrolled and analysed by WES. We also enrolled 78 patients and performed targeted-capture sequencing to confirm and extend the findings obtained by WES analysis. Thus, our cohort of CML-CP contained more patients who ultimately developed BC (48%, 71/148) compared to other cohorts, because we intentionally included paired CP and BC samples to investigate the molecular pathogenesis of the clonal evolution in CML. Detailed information on patients and institutions are described in Supplementary Table 1.
Ethics oversight	This study was approved by the institutional ethics committees of Kyoto University, Kobe City Medical Center General Hospital, Tokyo Medical University, Akita University Graduate School of Medicine, Juntendo University School of Medicine, Gifu University Hospital, Kurashiki Central Hospital, Hyogo College of Medicine, Dokkyo Medical University (Japan), the University of Tokyo, and Chang Gung Memorial Hospital-Linkou (Taiwan), and was performed in accordance with the Declaration of Helsinki. Informed consent was obtained from all participants.

Note that full information on the approval of the study protocol must also be provided in the manuscript.
